# Therapist reactions to patient personality: A pilot study of clinicians’ emotional and neural responses using three clinical vignettes from in treatment series

**DOI:** 10.3389/fnhum.2022.1037486

**Published:** 2022-11-28

**Authors:** Annalisa Tanzilli, Cristina Trentini, Alessandro Grecucci, Nicola Carone, Chiara Ciacchella, Carlo Lai, Miguel David Sabogal-Rueda, Vittorio Lingiardi

**Affiliations:** ^1^Department of Dynamic, Clinical Psychology, and Health Studies, Faculty of Medicine and Psychology, Sapienza University of Rome, Rome, Italy; ^2^Clinical and Affective Neuroscience Lab, Department of Psychology and Cognitive Sciences, University of Trento, Rovereto, Italy; ^3^Department of Brain and Behavioral Sciences, University of Pavia, Pavia, Italy; ^4^Department of Psychology, Catholic University of Sacred Heart, Milan, Italy

**Keywords:** therapist reaction, patient personality, neural response, ERPs, memory, hippocampus, LPPs, psychotherapy

## Abstract

**Introduction:**

Therapists’ responses to patients play a crucial role in psychotherapy and are considered a key component of the patient–clinician relationship, which promotes successful treatment outcomes. To date, no empirical research has ever investigated therapist response patterns to patients with different personality disorders from a neuroscience perspective.

**Methods:**

In the present study, psychodynamic therapists (*N* = 14) were asked to complete a battery of instruments (including the Therapist Response Questionnaire) after watching three videos showing clinical interactions between a therapist and three patients with narcissistic, histrionic/borderline, and depressive personality disorders, respectively. Subsequently, participants’ high-density electroencephalography (hdEEG) was recorded as they passively viewed pictures of the patients’ faces, which were selected from the still images of the previously shown videos. Supervised machine learning (ML) was used to evaluate whether: (1) therapists’ responses predicted which patient they observed during the EEG task and whether specific clinician reactions were involved in distinguishing between patients with different personality disorders (using pairwise comparisons); and (2) therapists’ event-related potentials (ERPs) predicted which patient they observed during the laboratory experiment and whether distinct ERP components allowed this forecast.

**Results:**

The results indicated that therapists showed distinct patterns of criticized/devalued and sexualized reactions to visual depictions of patients with different personality disorders, at statistically systematic and clinically meaningful levels. Moreover, therapists’ late positive potentials (LPPs) in the hippocampus were able to determine which patient they observed during the EEG task, with high accuracy.

**Discussion:**

These results, albeit preliminary, shed light on the role played by therapists’ memory processes in psychotherapy. Clinical and neuroscience implications of the empirical investigation of therapist responses are discussed.

## Introduction

The therapeutic relationship represents one of the most important mutative factors of good treatment outcome (Wampold, 2015; Norcross and Lambert, 2019). Of note, therapists’ emotional, cognitive, motivational, and behavioral response patterns to patients (in this context, we use the terms “therapist’s response” and “therapist’s reaction” interchangeably) play a crucial role in psychotherapy. Furthermore, they are a critical component of the patient–therapist relationship, which is strongly related to multifaceted processes involved in patient change (Gabbard, 1995; Gabbard and Westen, 2003; Hayes et al., 2018).

The theoretical-clinical roots of this relational dimension can be traced to the classical psychoanalytic concept of countertransference (Freud, 1910, 1912). It has been defined as the result of the patient’s influence on the analyst’s unconscious feelings or, in other words, the analyst’s transference on the patient. Stemming from unresolved psychological conflicts of the analyst, countertransference was originally considered an obstacle to the treatment of the patient. Later, in the 1950s, this overly restrictive perspective underwent a radical revision (cf., Heimann, 1950). According to the totalistic approach (Kernberg, 1965), countertransference was viewed as the wide range of feelings, thoughts, attitudes and behaviors experienced by the clinician in treating the patient; in these terms, it could be considered a valuable source of information about the patient’s intrapsychic and interpersonal dynamics. Therefore, the clinician reactions to a patient impact the diagnostic and therapeutic process, promoting a more accurate understanding of the patient’s psychological functioning, especially in the treatment of personality disorders (Beck et al., 2004; Dahl et al., 2014; Gabbard, 2014; Lingiardi and McWilliams, 2015; Yeomans et al., 2015; Bateman and Fonagy, 2016).

By definition, personality disorders are dysfunctional schemas of the self and the relationship between self and others. Patients’ styles of relating often emerge in the clinical relationship, when the therapist is drawn into interactions that reflect the patient’s enduring and maladaptive psychological and interpersonal dynamics (Bateman and Fonagy, 2006; Clarkin et al., 2006; Gabbard, 2014; Lingiardi and McWilliams, 2017). Accordingly, therapists’ recognition of their subjective reactions to a patient is important for their deep understanding of the patient’s relational patterns and inner experience.

Research has examined the association between therapists’ responses and patients’ personality syndromes, especially from the perspectives of clinicians and external observers. Some studies, based on the clinician’s perspective, have evaluated the quality and intensity of therapists’ emotional responses to patients presenting with DSM-IV (American Psychiatric Association [APA], 1994) axis II clusters (e.g., Betan et al., 2005; Røssberg et al., 2007), specific personality disorders (e.g., Bourke and Grenyer, 2013; Colli et al., 2014; Lingiardi et al., 2015; Tanzilli et al., 2017; Tanzilli and Gualco, 2020), and personality traits in the psychotherapy context (e.g., Røssberg et al., 2008; Tanzilli et al., 2018). Other empirical investigations, based on the observer’s perspective, have assessed therapists’ emotional experiences using vignettes or audio/video recordings of personality disordered patients (e.g., McIntyre and Schwartz, 1998; Schwartz et al., 2007). All of these studies have shown that cluster A disorders (i.e., paranoid, schizoid, and schizotypal personality disorders) are associated with disengaged response pattern; cluster B disorders (i.e., antisocial, borderline, histrionic, and narcissistic personality disorders) are correlated with overwhelmed/disorganized feelings, helplessness, hostility, withdrawal, and sexual attraction; and cluster C disorders (i.e., avoidant, dependent, and obsessive–compulsive personality disorders) are associated with nurturant and warm feelings. Moreover, these results have been consistent across therapists’ theoretical orientations. Overall, there is an increasing consensus that therapists’ awareness of their personal responses to patients may promote greater sensitivity in the diagnostic process, more accurate and clinically meaningful case formulations, and more effective therapeutic interventions (e.g., Gabbard, 2009a; Bateman and Fonagy, 2016; Lingiardi and McWilliams, 2017).

The present study adopted the totalistic approach (Kernberg, 1965) to explore which specific therapist reactions and neural responses allow clinicians to discriminate between patients with different personality disorders. To the best of our knowledge, no empirical research has ever examined therapists’ response patterns to patients from a neuroscience perspective. In addition, considering that previous studies have reported the influence of analyst gender on transference and countertransference responses (Berg et al., 2019; Chertoff et al., 2020), to prevent this effect from influencing the results, only male therapists were included in the present study. Specifically, the research aimed at contributing a preliminary empirical investigation of the clinical and neural responses—evaluated in terms of event-related potential (ERP) components—of psychodynamic clinicians while watching selected still images from videos of three patients (performed by actors) with various personality disorders (i.e., narcissistic, histrionic/borderline, depressive).

Event-related potentials (ERPs) are cortical measurements of the total electrical activity of postsynaptic potentials, which are generated in brain structures in response to sensory, cognitive, and motor events or stimuli (Peterson et al., 1995; Ghani et al., 2020). ERPs are collected *via* a non-invasive procedure, and they enable an advanced study of the temporal dynamics of stimulus processing (Luck, 2014). The components of the averaged ERP waveform indicate deflection (i.e., P for positive, N for negative), expected latency from the stimulus onset, and amplitude (i.e., neural resources required for processing) (Luck, 2005). ERPs that peak within the first 100 ms (approximately) after stimulus onset are known as sensory or exogenous, as they depend largely on the physical parameters of the stimulus; in contrast, later ERPs (emerging after 100 ms) are termed cognitive or endogenous, as they reflect the evaluation of information processing (Sur and Sinha, 2009; Sokhadze et al., 2017). Moreover, while early ERPs (i.e., N100, N200, P200) are mainly implicated in attention selection processes, subsequent ERPs (i.e., P300 or later) reflect stimuli evaluation or categorization.

The P1 component reflects early sensory processing within the extrastriate visual cortex (Vogel and Luck, 2000; Olofsson et al., 2008). It is typically larger in response to emotional stimuli (Carretié et al., 2004)—particularly faces (Holmes et al., 2007; Mueller et al., 2009; Mühlberger et al., 2009)—than non-emotional stimuli. The subsequent N100 component is a centro-parietal negative deflection, which peaks at approximately 130 ms after stimulus onset (Keil et al., 2001; Foti et al., 2009). Relative to P1, N1 reflects increased sensitivity to the emotional content of a visual stimulus, as it is larger for both pleasant and unpleasant stimuli, relative to neutral pictures (Keil et al., 2001; Carretié et al., 2007; Foti et al., 2009; Weinberg and Hajcak, 2011). P200 peaks at approximately 180 ms after stimulus onset (Carretié et al., 2004), and it is maximal at the anterior and central sites (Luck and Hillyard, 1994). This ERP component reflects the early affective evaluation and discrimination of visual stimuli (Begleiter et al., 1979; Conley et al., 1999) such as facial expressions (Eimer et al., 2003), as well as emotional words (Kissler et al., 2006; Kanske and Kotz, 2007). N2 is a fronto-central negativity that follows P2 and peaks at approximately 200–350 ms after stimulus onset. It is thought to be generated in the anterior cingulate cortex (ACC; Falkenstein et al., 1999; Pires et al., 2014). N2 is considered an index of cognitive control processes, as it deals with the inhibition of incorrect responses and is larger for conflict resolution tasks (Sokhadze et al., 2017; Ligeza et al., 2018). The subsequent broad centro-parietal positive deflection (i.e., P3) occurs between 300 and 600 ms after stimulus onset (Roche et al., 2004; Polich, 2007), and is thought to be generated by a more distributed network of cortical regions, relative to N2 (Polich, 2007; Foti and Hajcak, 2008; MacNamara and Hajcak, 2009, 2010). P3 amplitude is sensitive to motivationally salient stimuli, as it is modulated by both pleasant and unpleasant cues, regardless of whether the salience is defined in terms of the task demand or stimulus content (Olofsson et al., 2008; Hajcak et al., 2010; Weinberg and Hajcak, 2010); similar results have been reported for emotional words (Naumann et al., 1992) and faces (Allison et al., 1999; Grecucci et al., 2018; Sulpizio et al., 2019). Finally, the late positive potential (LPP) is a sustained positive deflection in the ERP waveform with centroparietal distribution, which emerges at approximately 300 ms following stimulus onset and persists for the duration of the stimulus (Schupp et al., 2000, 2004a; Foti et al., 2009) and beyond (for as long as 1,000 ms) (Hajcak and Olvet, 2008; Hajcak et al., 2010). LPP amplitude is larger for emotionally evocative stimuli (i.e., appetitive and aversive stimuli) than for neutral stimuli (Dunning and Hajcak, 2009; Hajcak et al., 2009), and it covaries with subjective arousal ratings of emotional stimuli (Cuthbert et al., 2000; Weinberg and Hajcak, 2010). Simultaneous functional magnetic resonance imaging–electroencephalogram (fMRI-EEG) studies have shown that LPP amplitude is also associated with activity in posterior cerebral regions (especially, lateral occipital, inferotemporal, and parietal visual areas), which are implicated in attention to and the perceptual processing of the motivational relevance of visual scenes (Sabatinelli et al., 2007). In the present research, three subcomponents of the LPP–hereafter termed late components (LC)–were identified according to their persistence: LC1 (i.e., 400–500 ms), LC2 (i.e., 500–700 ms), and LC3 (i.e., 700 ms).

In this study, supervised machine learning (ML)—known as a decision tree (Pekel and Özmen, 2020)—was applied (for the first time) to the domain of therapist responses. The advantage of a supervised ML approach (i.e., a branch of artificial intelligence) over standard frequentist approaches is that the algorithm extracts a mathematical function that maps one variable to another (e.g., ERP components to patients’ personality disorders), in order to predict new cases (Pekel and Özmen, 2020; Dadomo et al., 2022; Grecucci et al., 2022). Indeed, ML model performance refers to the prediction accuracy for new observations, rather than the degree to which certain factors explain the data.

Based on the above considerations, this pilot research aimed at evaluating whether: (1) therapists’ responses predicted which patient they observed and whether particular clinician reactions contributed to distinguishing between patients with different personality disorders; and (2) therapists’ ERPs predicted which patient they observed and, in particular, whether distinct ERP components allowed this forecast.

## Materials and methods

### Participants

The present study was conducted at the Department of Dynamic and Clinical Psychology, and Health Studies, Faculty of Medicine and Psychology, Sapienza University of Rome, in compliance with the Declaration of Helsinki (Helsinki, Finland, June 1964). A sample of 14 licensed therapists (*M* = 36.07; *SD* = 2.97; range 31–40) was recruited according to the following inclusion criteria: (a) self-identification as a cisgender man; (b) aged 30–40 years; and (c) reporting normal or corrected-to-normal vision. The exclusion criteria were: (a) the presence of neurological injury and psychiatric disease; (b) habitual drug or alcohol use; and (c) visual impairment. Participants self-declared their absence of psychiatric and neurological disease and use of drugs and alcohol. All clinicians provided written informed consent to participate and received no remuneration. The study was approved by the Department Research Ethics Committee (protocol number: 0000112/2020; date: 20/12/2020).

### Measures

*Clinical questionnaire*. It is an *ad hoc* clinician-report questionnaire that was used to obtain information about the therapists. Clinicians provided basic demographic and professional data, including their years of clinical experience.

*Shedler-westen assessment procedure-200* (SWAP-200; Westen and Shedler, 1999a,b; Shedler et al., 2014). The SWAP-200 is a psychometric system designed to provide a comprehensive assessment of personality and personality pathology. It consists of 200 items that clinicians sort into eight categories, ranging from 0 (not descriptive of the person) to 7 (most descriptive of the person), in order to comply with a fixed distribution. The SWAP–200 assessment furnishes: (a) a personality diagnosis, based on the matching of the patient assessment with 10 personality prototypes from the DSM–IV axis II (i.e., PD scales); (b) a personality diagnosis, based on the matching of the patient’s SWAP description with 11 empirically derived Q-factors/styles of personality; and (c) a dimensional diagnostic approach, based on a multifaceted model of personality pathology, including 12 clinically relevant dimensions [e.g., hostility, narcissism, emotional dysregulation, dysphoria, schizoid orientation; see Shedler and Westen (2004)]. The tool also includes a dimensional measure of psychological strengths and adaptive functioning. A personality disorder is assigned when scores on one or more PD scales and/or Q-factor or personality traits (in standardized T-scores) are ≥ 60 and the high-functioning scale score is < 60. The present study used only the SWAP-200 PD scales and personality dimensions, along with the healthy personality global functioning index (i.e., high-functioning scale). The SWAP-200 was designed for use by clinically experienced informants, and its reliability and validity have been extensively tested on different patient populations in several studies, including multi-observer studies comparing diagnoses by treating therapists with diagnoses by independent assessors, based on research interviews (e.g., Westen and Muderrisoglu, 2006; Blagov et al., 2012).

*Therapist response questionnaire* (TRQ; Zittel Conklin and Westen, 2003; Betan et al., 2005). The TRQ is a clinician-report instrument that assesses therapists’ emotional responses to patients. It consists of 79 items that measure a wide spectrum of thoughts, feelings, and behaviors expressed by therapists toward patients. Clinicians evaluate each item on a 5-point Likert scale ranging from 1 (not true) to 5 (very true). The present study used an empirically supported version of the TRQ (Tanzilli et al., 2016) to evaluate nine therapist response patterns: (a) helpless/inadequate, describing feelings of inadequacy, incompetence, and inefficacy; (b) overwhelmed/disorganized, describing intense feelings of overwhelm in response to the patient’s emotions and needs, as well as confusion, anxiety, or repulsion; (c) positive/satisfying, describing an experience of close connection, trust, and collaboration with the patient; (d) hostile/angry, describing feelings of anger, hostility, and irritation toward the patient; (e) criticized/devalued, describing a sense of being criticized, dismissed, or devalued by the patient; (f) parental/protective, describing a wish to protect and nurture the patient in a parental manner; (g) special/overinvolved, describing that the patient is very special, to the extent that the clinician may show some difficulty maintaining the boundaries of the therapeutic setting; (h) sexualized, describing the presence of sexual attraction toward the patient; and (i) disengaged, describing feelings of annoyance, boredom, withdrawal, or distraction during sessions. Scores for each scale are obtained by calculating the average score of the items comprising each factor. The nine TRQ factors showed excellent internal consistency (Streiner, 2003), obtaining the following Cronbach’s alpha values: criticized/devalued (α = 0.86), helpless/inadequate (α = 0.91), positive/satisfying (α = 0.84), parental/protective (α = 0.77), overwhelmed/disorganized (α = 0.88), special/overinvolved (α = 0.78), sexualized (α = 0.87), disengaged (α = 0.80), and hostile/angry (α = 0.87). The TRQ (and its version for adolescents, TRQ-A; Tanzilli et al., 2020) showed high reliability and validity in different clinical populations (cf., Stefana et al., 2020).

### Materials

#### Patient videos

Three videos were derived from the psychotherapy sessions depicted in the popular American television series In Treatment. Specifically, the videos showed clinical interactions between the therapist, Paul Weston, and three of his patients: Alex Prince, Laura Hill, and April (without surname). These three characters were chosen because they presented different personality pathologies (i.e., narcissistic, histrionic/borderline, depressive), and all of the clinical interactions between Paul (the therapist, played by an actor) and the three patients (performed by actors) clearly showed crucial aspects of psychological and interpersonal functioning related to these specific personality syndromes.

The selected contents of all three videos are reported below.

##### Alex

Alex is a military aircraft pilot. The video shows his initial exchange with Paul during their first psychotherapy session: Alex enters Paul’s office without introducing himself. He repeatedly asks whether Paul recognizes him, without explaining why. Alex tells Paul that he has collected information about Paul’s professional skills, and ultimately chose Paul because the data confirmed that “he was the best.” Alex reveals that he is accustomed to engaging with only the best. Thus, right from the first lines spoken, Alex shows an exaggerated sense of self-importance. He also appears dismissive and devaluing of Paul, interrupting him often and accusing him of not being a “good listener.” Alex structures and tries to dominate the interaction with Paul, leaving little room for the therapist, who feels cornered. Alex reveals that he is a war hero, and for that reason, he expected Paul to recognize him. In fact, he is the pilot who completed a delicate mission in Iraq that, by mistake, resulted in the death of many children. In the face of Paul’s disbelief, Alex emphasizes that he does not feel guilty. Rather, he proudly affirms that he did his duty with surgical precision, confirming the standards of excellence he had always guaranteed as one of the most qualified ‘top guns’ in a Navy special department.

##### Laura

Laura is an anesthesiologist who has been in a long-term relationship with another one of Paul’s patients, which seems to be progressing toward marriage. However, she is overwhelmed by her attraction to Paul. The clip begins in the middle of a conversation between Laura and Paul: she has just confessed her love to Paul and is openly disappointed by his reaction, having hoped that he would have felt the same. Laura details romantic and erotic fantasies about Paul’s positive response, trying to provoke his reaction. She also expresses frustration and anger toward Paul, pointing out how humiliating it is to see him do nothing after her declaration of love, and showing difficulty accepting rejection. At one point, Laura talks about a sexual encounter she had with a stranger in a club. She says that the reason she did not have sex with that man was not because she wished to remain faithful to her boyfriend, but because she was deeply in love with Paul. Continuing to provoke him, she describes in detail her fantasy of meeting Paul in a bar and being seduced by him. She then states that this is only her imagination. She knows nothing real can ever happen between them; however, she cannot resign herself to this reality. At the end of the clip, Laura appears desperate and lamenting, wondering what will become of her life and how she will manage this situation.

##### April

April is a young architecture student who has recently been diagnosed with lymphoma. In the clip, she never talks about her illness, but instead speaks about how she feels and how she has always felt in her life. April appears tired. Something about her eyes seems off and she feels depleted of strength. April tells Paul that she did not sleep a wink the previous night. Although she tries to make some jokes, her worry and sadness are evident. She describes her sleepless night as a nightmare and reveals that she sometimes considers hurting herself in order to end her excruciating pain. However, she quickly reassures Paul by saying that she is not serious, and that she would never do something so reckless. She is just trying to share her feelings with him. April talks about her fear of going crazy, recalling that she had this concern even when she was younger. However, she refuses to explore the topic further. She relates to Paul the thoughts that were troubling her the night before, and expresses—with a wistful attitude—her desire to return to a faraway place where she once felt safe. Paul asks her to describe this place in more detail, but April does not respond, and instead slumps down on the couch. She tells Paul to wake her up a little later, and then falls asleep.

##### Patient personality profiles

**FIGURE 1:**
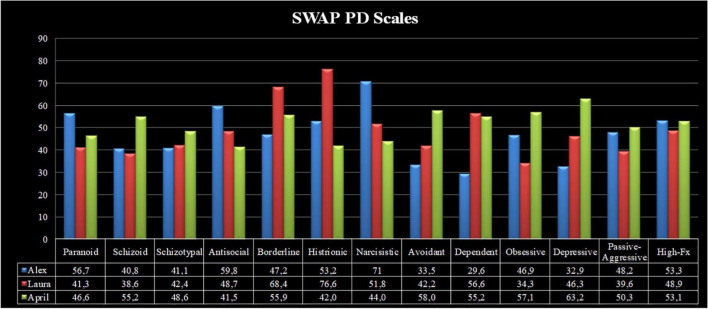
SWAP-200 personality profiles of Alex, Laura, and April.

**FIGURE 2:**
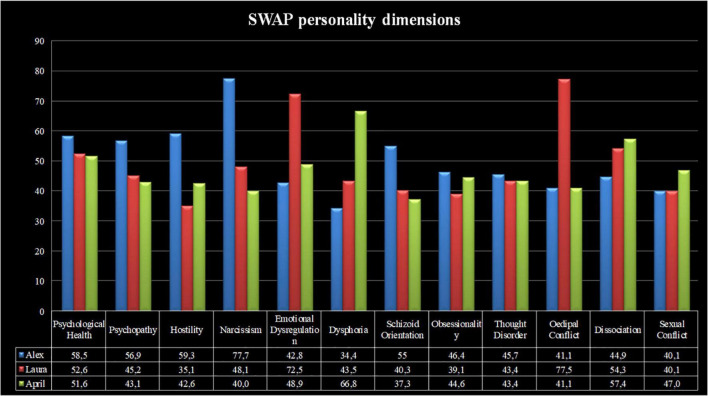
SWAP-200 personality dimensions of Alex, Laura, and April.

The personality syndromes of the abovementioned characters were assessed using the SWAP-200 (Westen and Shedler, 1999a; Shedler and Westen, 2004; Shedler et al., 2014), by two independent judges who blindly viewed the first three treatment sessions of each patient. The inter-rater reliability (IRR). was good (Spearman’s Rho = 0.75). Figures 1, 2 show the personality profiles of the three patients according to the SWAP-200 PD and personality dimension scales, respectively.

Considering the SWAP-200 PD scales, Alex presented with narcissistic personality disorder (T-score = 71), alongside clinically relevant antisocial and paranoid traits (T-scores = 59.8 and 56.7, respectively). His high-functioning score demonstrated an average level of overall personality functioning (T-score = 53.3). Laura presented with histrionic (T-score = 76.6) and borderline (T-score = 68.4) personality disorders, as well as strong dependent personality traits (T-score = 56.6). Her high-functioning score was slightly below average (T-score = 48.9). Finally, April showed a depressive personality disorder (T-score = 63.2), in addition to clinically relevant avoidant (T-score = 58) and obsessive (T-score = 57.1) personality traits. Her personality functioning was average (T-score = 53.1).

In terms of the SWAP-200 personality dimensions, Alex presented with high levels of narcissism (T-score = 77.7), along with clinically meaningful traits of hostility (T-score = 59.3) and psychopathy (T-score = 56.9). Laura showed a great degree of Oedipal conflict (T-score = 77.5) and emotional dysregulation (T-score = 72.5), whereas April presented with high levels of dysphoria (T-score = 66.8).

#### Visual stimuli

**FIGURE 3:**
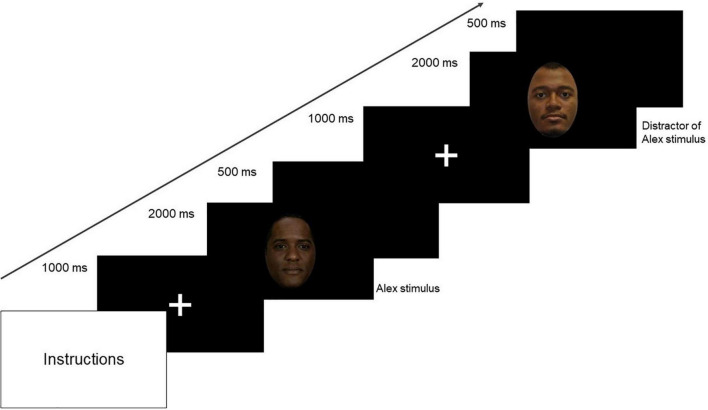
Schematic representation of the hdEEG experimental design.

The visual stimuli were comprised of 72 images of faces in color, with neutral emotional valence and a similar oval shape. Thirty-six still images depicting the patients’ faces (i.e., target stimuli; 12 for Alex, 12 for April, and 12 for Laura, respectively) were selected from the previously shown videos. Additionally, 36 images depicting unfamiliar faces (i.e., filler stimuli) were selected from the racially diverse affective expression (RADIATE) stimulus set (Conley et al., 2018), on the basis of their similarity to the three patients: 12 unfamiliar faces of African American men were chosen as Alex’s distractors, 12 unfamiliar faces of White women were chosen as April’s distractors, and 12 unfamiliar faces of White women were chosen as Laura’s distractors. During the visual stimuli task, the 72 images were randomly repeated for three times to get a total of 216 trials [36 trials *per* stimulus (Alex, April, Laura, distractor of Alex, distractor of April, and distractor of Laura]. Each trial started with a fixation cross displayed for 1,000 ms, followed by the visual stimulus (Alex, April, Laura, distractor of Alex, distractor of April, and distractor of Laura) presented for 2,000 ms. The trial ended with inter-stimulus interval (ISI) of 500 ms (Figure 3). The visual stimuli task lasted about 13 min. Given the specific experimental question (i.e., “Can we classify one patient versus the other?”), this paper does not report the analyses of filler stimuli. Rather, the present study focused on the faces of the real patients, to determine whether ERP components and therapist reactions could discriminate (i.e., classify, in ML terms) between each pair of patients (i.e., Alex vs. April, Alex vs. Laura, and April vs. Laura).

### Experimental procedure

Therapists were asked to complete a battery of instruments (including the clinical questionnaire and the TRQ; see “Measures” section) after watching the three video clips described above. Each participant filled out the questionnaires in consideration of how they might feel when interacting with the patients observed in the videos. To prevent any order effect, videos were shown to participants in a random order. Subsequently, clinicians were asked to complete a laboratory experiment in which they viewed a series of still images from the videos. All participants performed the visual task in a dimly lit room, seated at a distance of 80 cm from the PC monitor displaying the stimuli (27 cm, 75 Hz, 1,024 × 768). The visual stimuli were presented using E-Prime software (v.2.0.8.90; Psychology Software Tools), and the high-density electroencephalography (hdEEG) signal of each participant was recorded during the task. To limit potential biases associated with preconceived stereotypes about personality syndromes, participants were unaware of patients’ personality diagnoses. Following the experimental procedure, clinicians were asked to assign a personality diagnosis to all three patients based on the observed video material. The reliability of their responses was about 90%.

### High-density electroencephalography recordings and data processing

HdEEG signals were recorded continuously at 250 Hz with reference to the vertex (Cz), with impedances kept below 50 kΩ, using a 256-channel Hydrocel Geodesic Sensor Net and Net Station software (v.4.4.2; Electrical Geodesic, Inc., Eugene, OR, USA). Subsequently, hdEEG data were digitally filtered at 30 Hz low-pass in offline mode. Data for each participant were segmented in epochs of 1,100 ms, ranging from 100 ms before to 1,000 ms after stimulus onset. Artifact detention was set to 200 μV for bad channels (i.e., noisy electrodes), 140 μV for eye blinks, and 100 μV for electrodes detecting eye movements (Picton et al., 2000; Bourisly and Shuaib, 2018; Lai et al., 2020; Altavilla et al., 2021). Segments containing eye blinks, eye movement, or more than 15 bad channels were excluded. A baseline correction of −100 ms before stimulus onset was applied. The amplitude and the latency of ERP components in response to each type of stimulus (Alex, April, and Laura) were extracted automatically through Net Station software, averaging the clean segmented trials for each participant. In the study, the following intervals were set: 80–160 ms for P100; 160–220 ms for N170; 270–400 ms for P300; 400–500 for LC1; 500–700 for LC2; and 700–1,000 for LC3. Following hdEEG signal cleaning from artifacts, as reported in previous studies (Picton et al., 2000; Tanner et al., 2015; Lai et al., 2020; Altavilla et al., 2021), the following electrode locations were chosen for each montage: occipital (O1 and O2), occipito-temporal (left: 85, 96, 107, 108, 109; right: 151, 160, 161, 171, 172), parietal (left: 77, 78, 79, 86, 87, 88; right: 142, 143, 153, 154, 162, 163), and frontal (left: 23, 24, 30, 35, 42; right: 6, 7, 206, 215, 224).

Data were analyzed for peak amplitudes and latencies at P100 on the occipital and occipito-temporal montages; and at N170 on the occipital, occipito-temporal, and parietal montages. Moreover, data were analyzed for mean amplitudes and latencies at P300 on the occipito-temporal, parietal, and frontal montages. Lastly, at LC1, LC2, and LC3, data were analyzed for mean amplitudes of the occipito-temporal, parietal, and frontal montages.

### Source analysis (sLORETA)

To locate the neural generators of the ERP components, hdEEG signals were processed using the standardized low-resolution electromagnetic tomography (sLORETA) (Pascual-Marqui, 2002) inverse model of the GeoSource software (v.2.0; EGI, Eugene, OR, USA), with Tikhonv 1 × 10^–4^ regularization. sLORETA assumes the current density standardization, which considers not only the variance of noise in the hdEEG measurements but also the biological variance in the actual signal (Pascual-Marqui, 2002). Biological variance is thought to be independent and uniformly distributed in the brain, resulting in a linear image localization with exact and zero localization error (Jatoi et al., 2014). Source locations were derived from the probabilistic map of the Montreal Neurological Institute 305 subjects (i.e., MNI305 average). On this basis, gray matter volume was parcellated into 7-mm voxels. Each voxel served as a source location with three orthogonal orientation vectors, resulting in a total of 2,447 source triplets, whose anatomical labels were estimated using a Talairach Daemon (Lancaster et al., 2000; Cecchini et al., 2013; Massaro et al., 2018). Magnetic resonance imaging normalization and data extraction were performed for each participant, and the mean intensities of specific Brodmann areas (BAs) were extracted for each ERP component.

In accordance with the hypotheses and the literature regarding the neurobiological correlates of the visual processing of faces (Eimer and Holmes, 2007; Vuilleumier and Pourtois, 2007; Hung et al., 2010; Lai et al., 2020), the following regions of interest (ROIs) were chosen for the sLORETA analyses: occipital, limbic, anterior cingulate cortex, posterior cingulate cortex, parietal, temporal, and prefrontal cortex. For each ROI, the following BAs in both hemispheres were selected: BA17, BA18, and BA19 for the occipital ROI; amygdala, insula, amygdala-hippocampus junction, and hippocampus for the limbic ROI; BA24, BA32, and BA33 for the anterior cingulate cortex ROI; BA23, BA30, and BA31 for the posterior cingulate cortex ROI; BA01, BA02, BA03, BA05, and BA07 for the parietal ROI; BA20, BA 21, BA 22, BA 37, BA 38, BA 41, BA 42, and BA 43 for the temporal ROI; and BA09, BA10, BA11, BA46, and BA47 for the prefrontal cortex ROI. The mean intensity of each BA in response to the target stimuli (i.e., Alex, April, Laura) was extracted for each ERP component (i.e., P100, N170, P300, LC1, LC2, LC3).

### Statistical analysis

Statistical analyses were performed in JASP (v.016; JASP Team 2021), using a decision tree classification model. The decision tree is a supervised ML algorithm that obtains predictive estimates for variables that take discrete values (Loh, 2011). Tree models in which the target variable has a discrete value are called classification trees. Such trees are structured so that every leaf represents a class label (i.e., Alex, Laura, April), and branches represent relevant features that predict class labels (i.e., therapist reaction, ERP components). The algorithm finds the optimal decision tree by computing the error between the predicted value and the actual value at each split point. Split point errors are then compared across variables and the lowest prediction error is used to generate the tree (Pekel and Özmen, 2020). Such split points indicate the most relevant predictive features.

In the present study, the data split was set to use 20% of the data: of the 28 observations (i.e., 14 therapist responses for two patients at a time) 23 were used for model training and five were used for model testing. The hold-out method was used to test the predictive value of each model, with the following split: 80% of observations were used to train the model, and the remaining 20% were used to test the model (i.e., 23 observations were used for training and five were used for testing). The algorithmic settings were as follows: the minimum number of observations for a split was set to 20, the minimum number of observations in the terminal was set to 7, the max iteration depth was set to 30; and the complexity parameter was set to 0.01. Predictors were scaled. Models were iterated five times and the best model was selected on the basis of the highest accuracy (i.e., bake-off method).

## Results

### Characteristics of therapists’ responses to patients

**FIGURE 4:**
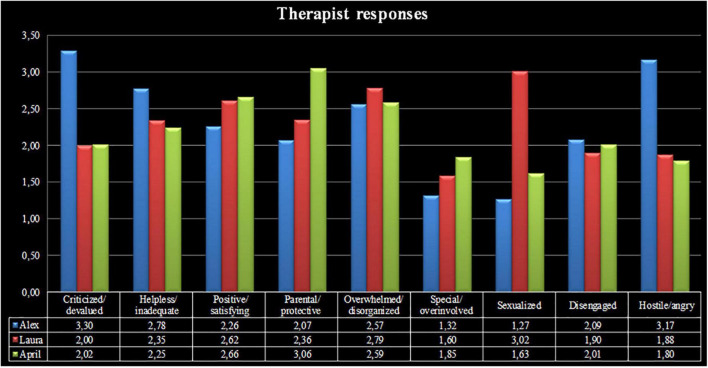
Therapist responses to Alex, Laura, and April.

Figure 4 depicts the therapist responses to patients with narcissistic, histrionic/borderline, and depressive personality disorders (i.e., Alex, Laura, and April, respectively). The mean scores of the TRQ patterns indicated that the strongest therapist response patterns elicited by Alex were criticized/devaluated (*M* = 3.30, *SD* = 0.62) and hostile/angry (*M* = 3.17, *SD* = 0.87), whereas the most intense therapist reactions patterns evoked by Laura and April were sexualized (*M* = 3.02, *SD* = 0.97) and parental/protective (*M* = 3.06, *SD* = 0.97), respectively.

### Prediction of patient personality disorder from therapists’ responses and ERP components

The results of the decision tree are reported below for each pair of patients.

The first comparison focused on the therapist reactions to the narcissistic (i.e., Alex) versus the depressive (i.e., April) patient. The decision tree performance when classifying Alex versus April achieved a classification accuracy of 100%. Of note, the precision (i.e., positive cases predicted) was 100% and the recall (i.e., true positive rate) was 100%. The area under the curve was 1.0.

**FIGURE 5:**
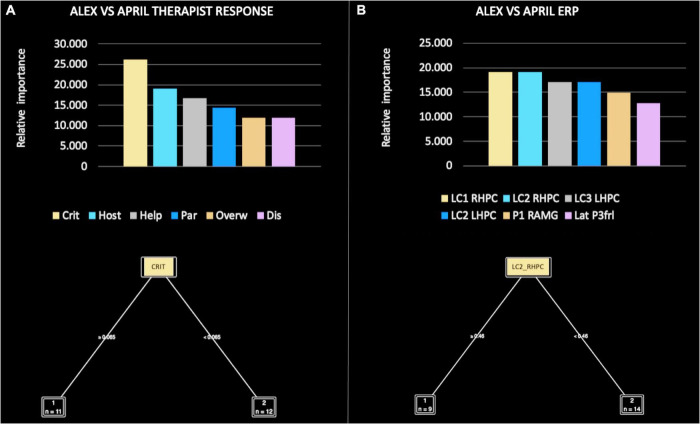
Relative importance of all therapist response patterns **(A)** and event-related potential (ERP) components **(B)**, included in the machine learning (ML) model for Alex versus April.

The relative importance of all therapist response patterns included in the ML model were, in order: criticized/devalued (26.190), hostile/angry (19.048), helpless/inadequate (16.667), parental/protective (14.286), special/overinvolved (11.905), and disengaged (11.905) (Figure 5A). Of note, the criticized/devalued pattern was responsible for the main split between Alex and April, according to the following rule: if criticized/devalued ≥ 0.065 then Alex, if criticized/devalued < 0.065 then April.

In the comparison focused on ERP components, the decision tree performance when classifying Alex versus April achieved a classification accuracy of 80%. Thus, the extracted function was able to accurately guess which patient the therapist was observing at a level far above chance. The precision (i.e., positive cases predicted) was 85% and the recall (i.e., true positive rate) was 80%. The area under the curve was 0.75%.

The relative importance of ERP components was, in order: LC1 RHPC (19.149), LC2 RHPC (19.149), LC3 LHPC (17.021), LC2 LHPC (17.021), P1 RAMG (14.894), and LatP3 frL (12.766) (Figure 5B). Of note, LC1 RHPC was responsible for the main split between Alex and April, according to the following rule: if LC1 RHPC ≥ 0.099 then Alex, if LC1 RHPC < 0.099 then April.

The second comparison focused on the therapist responses to the narcissistic (i.e., Alex) versus the histrionic/borderline (i.e., Laura) patient. The decision tree performance when classifying Alex versus Laura achieved a classification accuracy of 80%. The precision (i.e., positive cases predicted) was 87% and the recall (i.e., true positive rate) was 80%. The area under the curve was 0.83.

**FIGURE 6:**
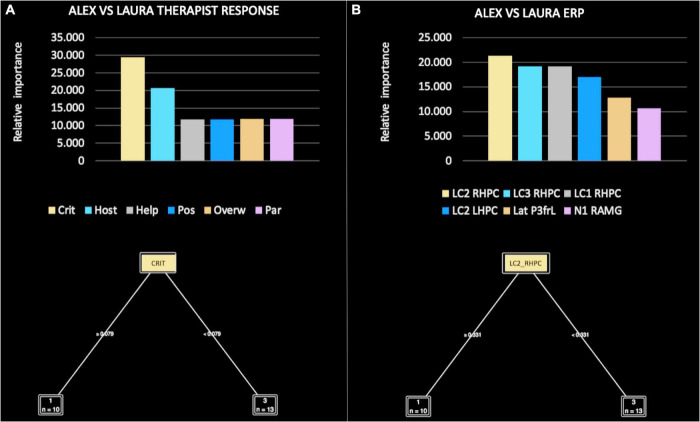
Relative importance of all therapist response patterns **(A)** and event-related potential (ERP) components **(B)**, included in the machine learning (ML) model for Alex versus Laura.

The relative importance of therapist responses was, in order: criticized/devalued (29.412), hostile/angry (20.588), helpless/inadequate (11.765), positive/satisfying (11.765), overwhelmed/disorganized (11.905), and parental/protective (8.824) (Figure 6A). Of note, the criticized/devalued pattern was responsible for the main split between Alex and Laura, according to the following rule: if criticized ≥ 0.079 then Alex, if criticized < 0.079 then Laura.

In the comparison focused on ERP components, the decision tree performance when classifying Alex versus Laura achieved a classification accuracy of 80%. Thus, the extracted function was able to accurately guess which patient the therapist was observing at a level far above chance. The precision (i.e., positive cases predicted) was 85% and the recall (i.e., true positive rate) was 80%. The area under the curve was 0.75.

The relative importance of ERP components was, in order of importance: LC2 RHPC (21.277), LC3 RHPC (19.149), LC1 RHPC (19.149), LC2 LHPC (17.021), LatP3 frL (12.766), and N1 RAMG (10.638) (Figure 6B). Of note, LC2 RHPC was responsible for the main split between Alex and Laura, according to the following rule: if LC2 RHPC ≥ 0.331 then Alex, if LC3 < 0.331 then Laura.

The third comparison focused on the therapist reactions to the depressive (i.e., April) versus the histrionic/borderline (i.e., Laura) patient. The decision tree performance when classifying Alex versus April achieved a classification accuracy of 100%. The precision (i.e., positive cases predicted) was 100% and the recall (i.e., true positive rate) was 100%. The area under the curve was 1.0.

**FIGURE 7:**
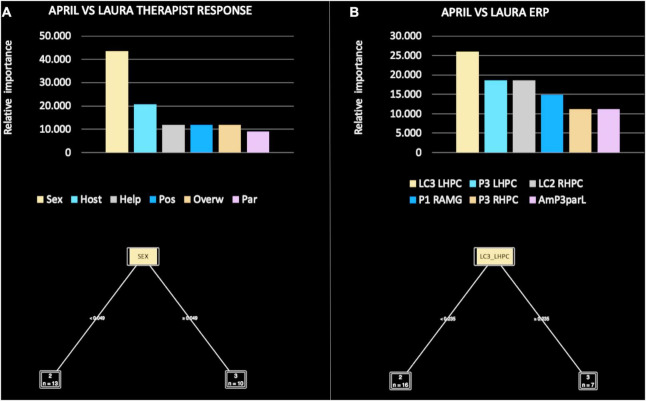
Relative importance of all therapist response patterns **(A)** and event-related potential (ERP) components **(B)**, included in the machine learning (ML) model for April versus Laura.

The relative importance of therapist reactions was, in order: sexualized (43.478), overwhelmed/disorganized (21.739), parental/protective (13.043), helpless/inadequate (8.696), hostile/angry (8.696), and criticized/devalued (4.348) (Figure 7A). Of note, the sexualized pattern was responsible for the main split between April and Laura, according to the following rule: if sexualized ≤ 0.049 then April, if LC3 > 0.049 then Laura.

In the comparison focused on ERP components, the decision tree performance when classifying April versus Laura achieved a classification accuracy of 100%. The precision (i.e., positive cases predicted) was 100% and the recall (i.e., true positive rate) was 100%. The area under the curve was 1.0.

The relative importance of ERP components was, in order: LC3 LHPC (25.926), P3 LHPC (18.519), LC2 RHPC (18.519), P1 RAMG (14.815), P3 RHPC (11.111), and AmP3 parL (11.111) (Figure 7B). Of note, LC3 LHPC was responsible for the main split between April and Laura, according to the following rule: if LC3 ≤ 0.235 then April, if LC3 > 0.235 then Laura.

## Discussion

The main aims of the present pilot study were to investigate, through an experimental research design: (1) whether therapists’ specific emotional, cognitive, motivational, and behavioral responses to visual stimuli depicting three patients with different personality disorders distinguished between patients in a statistically systematic and clinically meaningful way; and (2) whether therapists’ distinct neural responses (i.e., ERP components) while viewing visual stimuli of patients’ faces during the EEG task differentiated between the three patients, with high accuracy. An ML method was adopted to evaluate the ability of a model (consisting of clinical or neural variables) to accurately predict the facial stimulus of a personality-disordered patient from that of another patient (using pairwise comparisons) during the EEG experimental procedure.

Regarding the first goal, the general question posed was: “Is it possible to predict which patient the therapist observed from their subjective reactions? If so, which pattern of therapist response better predicts one patient than another?” Overall, the findings confirmed that therapists’ reactions to visual stimuli depicting patients’ faces during the EEG task precisely discriminated between patients presenting with narcissistic (i.e., Alex), histrionic/borderline (i.e., Laura), and depressive (i.e., April) personality disorders. In other words, specific therapist responses were associated with the facial stimuli of patients with particular personality pathologies, in a coherent and predictable manner.

In more detail, the ML models comparing Alex and April (Figure 5A), Alex and Laura (Figure 6A), and April and Laura (Figure 7A) distinguished the visual stimuli of different patients on the basis of clinician reactions with high accuracy (ranging from 80 to 100%). Of note, the criticized/devalued therapist reaction emerged as the most important predictor, with the strongest discriminatory power in identifying the narcissistic patient (i.e., Alex) from the depressive and histrionic/borderline patients (i.e., April and Laura, respectively). Conversely, the sexualized reaction was the most relevant and clinically useful relational dimension in comparing April and Laura, with a cut-off value that maximized both accuracy and the specificity of prediction.

These results are supported by a wide corpus of clinical observations (e.g., Gabbard, 2014; Yeomans et al., 2015; Lingiardi and McWilliams, 2017) and empirical evidence in the field (Betan et al., 2005; Dahl et al., 2012, 2014; Bourke and Grenyer, 2013; Colli et al., 2014; Nissen-Lie et al., 2022). In this vein, previous studies investigating the relationship between patients’ narcissistic personality disorder and therapists’ responses in the psychotherapy context (e.g., Betan et al., 2005; Ronningstam, 2012, 2016; Tanzilli et al., 2017) have highlighted that narcissistic patients typically evoke negative emotional reactions in clinicians, potentially disrupting their ability to benefit from the clinical treatment (McWilliams, 2004). Clinicians tend to feel devaluated, unappreciated, demeaned, or belittled by narcissistic patients during psychotherapy (e.g., Kernberg, 1975, 2014; Gabbard, 2009b). These reactions may reflect patients’ typical affective-interpersonal difficulties, which frequently involve behavior that is domineering, controlling, competitive, hostile, and cold, as well as a defensive tendency to criticize and devalue others due to feelings of inferiority and attempts to stabilize fluctuating self-esteem (e.g., Perry and Perry, 2004; Clemence et al., 2009; Ogrodniczuk and Kealy, 2013).

Other empirical investigations have found that borderline patients tend to elicit therapist response patterns characterized by feelings of overwhelm, helplessness, and overinvolvement (e.g., Lingiardi et al., 2015). These reactions may reflect patients’ severe emotional dysregulation and contradictory self and other representations, which are related to the overuse of primitive defenses such as spilling and projective identification (e.g., Clarkin et al., 2006). Moreover, histrionic patients tend to evoke a sexualized response in therapists (Gabbard, 2014), perhaps in association with their tendency to display seductive attitudes in their relationships with others (including the clinician). According to the clinical literature (e.g., McWilliams, 2004), this erotization may have a defensive function, serving toward off feelings of weakness or a fear of intimacy in therapy, or to maintain a sense of control and power over the therapist.

Finally, research has shown that depressive patients mainly elicit positive and warm feelings in the therapist (e.g., Blatt, 2004; Blatt and Shahar, 2004; Hennissen et al., 2019; cf. also McWilliams and Shedler, 2017). In general, such patients are cooperative in the therapeutic relationship; accordingly, therapists’ nurturant and protective feelings toward depressive patients may relate to their collaborative attitudes. Moreover, depressive patients are characterized by chronic vulnerability to painful affect (especially depression, guilt, shame, and perceived inadequacy), which may provoke in clinicians a strong impulse to care, even to the point of overinvolvement (cf., McWilliams and Shedler, 2017).

Regarding the second goal, the research question was: “Is it possible to predict which patient therapists observed from their brain states (in terms of ERPs)? If so, what are the main ERP components that contribute to this predictive model?” Overall, the findings showed that LPP amplitude in the hippocampus was able to reliably discriminate between patients. The LLP is conceived of as a neural index of the controlled attentional processes (Schupp et al., 2004b; Hajcak et al., 2009; Frenkel and Bar–Haim, 2011) involved in the affective appraisal of stimuli (Schupp et al., 2006; Wessing et al., 2013), and particularly emotional faces.

It has been largely documented that, during facial processing, information from the structural features of the face is rapidly integrated with (and affected by) contextual variables, derived from the observed face (e.g., eye gaze), its surrounding body elements (e.g., body posture), the external scene (e.g., visual background), and the perceiver (e.g., his/her biographical knowledge or processing biases) [(Adams and Kleck, 2003, 2005; Artuso et al., 2021); for a review see Meeren et al. (2005), Wieser and Brosch (2012), Rischer et al. (2020)].

(Adams and Kleck, 2003, 2005; Artuso et al., 2021) for a review see Meeren et al. (2005), Wieser and Brosch (2012), Rischer et al. (2020). Numerous studies have shown that this context effect on facial processing is limited to late latencies (Bradley, 2009; Hajcak et al., 2009, 2010; Diéguez–Risco et al., 2013; Wessing et al., 2013), and that contexts that are conditioned to arousal (particularly threat) increase LPP amplitude (Klein et al., 2015; Xu et al., 2016; Stolz et al., 2019). Of note, these findings disconfirm those of less robust investigations reporting the neural processing of facial expressions and contextual variables as occurring at earlier latencies, involving the vertex positive potential (VPP) and its negative counterpart (N170) (Righart and De Gelder, 2006, 2008; Hietanen and Astikainen, 2013; Diéguez-Risco et al., 2015).

A large body of research has shown that the LPP is also a specific index of memory encoding and storage (Palomba et al., 1997; Schupp et al., 2000), particularly when individuals are confronted with emotionally arousing stimuli (Mecklinger and Pfeifer, 1996; Azizian and Polich, 2007). In line with this, it has been found that meaningful and arousing stimuli elicit larger LPPs and are better retrieved than neutral and relatively less arousing stimuli (Palomba et al., 1997; Dolcos and Cabeza, 2002).

How do emotionally arousing stimuli facilitate memory encoding and storage? Compared to the stimulus valence (i.e., unpleasant–pleasant), which influences relatively early components (100–250 ms), stimulus arousal level (i.e., low–high) influences relatively later (200–1,000 ms) components (Olofsson and Polich, 2007). Moreover, while stimulus valence is indicative of initial selective attention toward salient image content [with unpleasant stimuli producing stronger emotional effects than pleasant stimuli; for a review, see Olofsson et al. (2008)], stimulus arousal leads to an increase in attentional resources that, in turn, facilitates memory encoding and storage (Dolcos and Cabeza, 2002; Schupp et al., 2004a,b).

In the present research, LPP amplitude proved more sensitive in identifying the facial stimuli of patients with different personality pathologies. However, some slight differences merit further discussion. Overall, consistent with the research mentioned above, it may be assumed that LPPs are not merely elicited by patients’ faces, *per se*. Instead, they might indicate that, during the EEG task, therapists were engaged in: (a) the attentive processing of affectively meaningful stimuli and “contextual variables” (e.g., Schupp et al., 2006; Bradley, 2009; Hajcak et al., 2009, 2010; Diéguez–Risco et al., 2013; Wessing et al., 2013) related to patients with distinct personality disorders who were previously observed in the videos, and (b) the memory encoding of patients’ (emotionally arousing) faces (Mecklinger and Pfeifer, 1996; Schupp et al., 2000; Azizian and Polich, 2007).

In more detail, the ML models found that amplitudes of specific LPP sub-components were able to accurately discriminate between the facial stimuli of patients with different personality pathologies. Of note, the earliest LPP sub-component (LC1) occurred when therapists were confronted with the facial stimuli of a narcissistic (i.e., Alex) versus a depressive (i.e., April) patient (Figure 5B); the middle LPP sub-component (LC2) occurred when therapists were confronted with the facial stimuli of a narcissistic (i.e., Alex) versus a histrionic/borderline (i.e., Laura) patient (Figure 6B); and the last—and most persistent—LPP sub-component (LC3) occurred when therapists were confronted with the facial stimuli of a depressive (i.e., April) versus a histrionic/borderline (i.e., Laura) patient (Figure 7B).

According to the abovementioned studies on therapist responses to personality-disordered patients, some considerations should be addressed. Narcissistic patients tend to evoke very negative and intense therapist reactions, characterized by hostility, irritation, and contemptuous derogation; histrionic/borderline patients mostly elicit a strong sense of incompetence, inadequacy, confusion, and sexual arousal; whereas depressive patients evoke positive reactions (i.e., nurturing, protection, care). As depicted in Figure 4, the therapist reactions to Alex, Laura, and April were consistent with the empirical literature, suggesting that patients with narcissistic and histrionic/borderline diagnoses show “more arousing” features than depressive patients. Noteworthy, narcissistic and histrionic/borderline diagnoses are among the most challenging clinical syndromes to treat in psychotherapy, especially due to the difficulty of establishing a “good enough” therapist–patient relationship (e.g., Ogrodniczuk and Kealy, 2013; Gabbard, 2014; Caligor et al., 2015; Yeomans et al., 2015).

From this perspective, we may assume that the involvement of the earliest LC1 and LC2 ERP sub-components when therapists were confronted with the facial stimuli of Alex and Laura, respectively (compared with the last and more persistent LC3 sub-component predicting April’s face), reflects the particular personality characteristics of these patients. In other words, during the EEG procedure, clinicians’ attention processes seem to have been engaged earlier when confronted with (emotionally arousing) visual stimuli connected to Alex and Laura. Conversely, when therapists confronted April’s (less arousing) stimuli, their attention and affective meaning attribution processes required later latencies. If future research is able to corroborate these preliminary results, we could argue for the great clinical utility of LPP sub-components in discriminating between patients with distinct affective and interpersonal characteristics.

The present results also showed that, in each tested pair of patients, the LPP was maximally localized in the hippocampus. This suggests that this cerebral region plays a relevant role in memory processing (Cohen and Eichenbaum, 1995; Eichenbaum, 2001) and the recall of rich and detailed memories (i.e., episodic memories) (e.g., Scoville and Milner, 1957; Addis et al., 2004; Barron et al., 2020). The hippocampus is conceived of as the apex of cortical processing, allowing pattern-separated information to be rapidly bound to support the recall of episodic memories (Felleman and Van Essen, 1991; Mishkin et al., 1998; Addis et al., 2007; Montaldi and Mayes, 2010; Mesulam, 2013). In more detail, the hippocampus receives sensory cortex inputs (including information from all sensory modalities) from the entorhinal, perirhinal, and parahippocampal cortices. On this pathway to the hippocampus, sensory inputs become increasingly elaborated (Pandya and Seltzer, 1982; Van Essen and Maunsell, 1983), and make broad cortico-cortical connections that allow the hippocampal output to be projected back to the neocortex (Witter et al., 1989; Witter, 1993; Squire et al., 2004).

At a physiological level, memory recall implies that, in the presence of an actual retrieval cue, the hippocampal representation of the original experience is promptly reactivated (Wheeler et al., 2000; Rugg and Vilberg, 2013). It has been suggested that, by virtue of reciprocal anatomical connectivity, the hippocampus may coordinate (through excitatory actions) activity in neocortical circuits (Barron et al., 2020), causing the reinstatement of the original neocortical pattern and triggering a re-experience of the original event (Eldridge et al., 2000; Maguire et al., 2001; Moscovitch and McAndrews, 2002; Daselaar et al., 2008; Rugg and Vilberg, 2013).

Of note, beyond sensory information about the external world, interoceptive information regarding the internal and motivational state of the organism from subcortical and brainstem systems is also transmitted to the hippocampus (Strange and Dolan, 2006). It has been proposed that, during memory recall (and particularly during autobiographical memory recall), specific qualities associated with memories (e.g., vivid details, emotionality, personal significance) exert a modulating influence on hippocampal activity and thereby the re-experience of past events (Wheeler et al., 1997; Strange and Dolan, 2006). These assumptions are coherent with the emotional context maintenance and retrieval model (eCMR; Talmi et al., 2019), which extends the original context maintenance and retrieval model (CMR; Polyn et al., 2009) to the emotional domain. In line with this, the eCMR assumes that memory retrieval is not only influenced by the conditions of the retrieval, itself, but also by the qualities of memories with a personal, emotional meaning (Talmi et al., 2019). In summary, the eCMR provides a conceptual explanation of what we need (or are prone) to remember. The hippocampus plays a central role in these processes, given its connectivity with neocortical areas involved in declarative memory (particularly dorsal anterior cingulate regions; Bush et al., 2000; Critchley, 2004) and other cerebral areas (e.g., the amygdala as well as broad networks underlying attentional and contextual processing) that favor the enhancement of memory for emotional stimuli, during both encoding and retrieval (see Richardson et al., 2004; Dolcos et al., 2012). In light of this evidence, it is plausible to assume that, while observing patients, therapists not only retrieve memories of those patients (and their specific personality disorders), but they also engage attentional and emotional resources to integrate such memories with those of their previous clinical experience with personality disordered patients.

What impact might therapists’ previous clinical experience with patients reporting specific personality disorders (e.g., in this case, narcissistic, histrionic/borderline, depressive) have on “real” patients with similar personality diagnoses, such as Alex, Laura, and April? There is mounting empirical evidence to suggest clear connections between memory processes, psychopathology, and psychotherapy (see, e.g., the work of Lane et al., 2015 and all related commentaries), neglecting the ways in which therapists remember or, in other terms, how they use attention and memory processes to serve a healing function [e.g., Gazzaniga, 2008; Boston Change Process Study Group, 2010; for a deeper discussion, see Ekstrom (2014)].

To our knowledge, no prior study has focused on clinicians’ memory processes; consequently, based on the extensive neuroscience literature reported above, we could only speculate that, in our pioneering investigation, therapists’ meaningful memory recall processes (regarding memories of both the observed patients and previous clinical experiences) affected their ability to distinguish between the facial stimuli of patients with different personality disorders during the EEG procedure. As Addis et al. (2007) underlined, “events in one’s past and future are inherently personal and thus should be comprised of autobiographical information” (p. 2). From this premise, and considering the hippocampal role in the reintegration of recollective details in autobiographical memory, Addis et al. suggested that this cerebral structure may also “bind event details for novel future scenarios” (p. 2). This assumption has been repeatedly confirmed by neuroscience research (Okuda et al., 2003; Addis et al., 2007; Rugg and Vilberg, 2013). In fact, the hippocampus comprises part of a common neural network—as well as the medial prefrontal cortex (MPFC) and medial parietal cortex (MPC), extending into the retrosplenial cortex and precuneus (Okuda et al., 2003; Addis et al., 2007; Addis and Schacter, 2008)—that is engaged when both remembering the past and imagining the future.

It has been demonstrated that, in the reconstruction of a past event or the creation of a future event, the hippocampus is engaged early (Addis et al., 2007). Such engagement even precedes that of the PFC, which is known to process self-referential information (Craik et al., 1999; Gusnard et al., 2001; Johnson et al., 2002) such as autobiographical memories (Maguire, 2001; Gilboa, 2004) and imagined future events (Okuda et al., 2003). According to the constructive episodic simulation hypothesis (Schacter and Addis, 2007), the common neural network for past and future events reflects a reliance on memory to collect details that may be coherent with both past and imagined representations.

Of note, it has also been shown that hippocampal activity is higher when future—as opposed to past—events are imaged (Okuda et al., 2003; Addis et al., 2007). Although both past and future events engage a common neural network aimed at retrieving information from memory, only future scenarios require previously bound information (i.e., memory traces) to be searched, collected, and re-integrated into a novel future event (Cohen et al., 1999; Eichenbaum, 2001). This implies that additional hippocampal resources are needed to allow the details of past events to be successfully bound into a coherent event (Schacter and Addis, 2007).

This relevant role of the hippocampus allows us to extend the clinical implications of our preliminary findings. If processes involving the hippocampus (along with the temporal lobe and other cortical regions) link the past with the present and allow for projection into the future, it may be reasonable to speculate that: (a) therapists’ episodic and autobiographical memories (strongly connected to their clinical practice and emotionally significant experiences) exert a modulatory effect on attention processes and the attribution of personal meaning to visual stimuli depicting patients (through complex mechanisms of encoding, storage, and retrieval); and (b) clinicians are able to integrate this information and use semantic knowledge involving personally meaningful representations and symbols to establish accurate patient diagnoses, develop “good enough” therapeutic alliances, and plan effective treatment interventions (Ekstrom, 2014).

The convergence of several empirical and clinical contributions suggests that therapist reactions to narcissistic patients may provoke enactments of judgment, harsh commentary, premature interpretation, criticism, and/or accusatory statements (Gabbard, 2009b; Ronningstam, 2016; cf. also Crisp and Gabbard, 2020); whereas the treatment of histrionic/borderline patients may require firm boundaries to be maintained in the therapeutic relationship, in order to allow for a safe and stable context for patients’ self-examination and interpretation of their emotional and relational difficulties (McWilliams and Shedler, 2017). Conversely, when working with depressive patients, the clinical challenge may be the development of an “idealized” patient-therapist relationship in which there is no space for the disappointment and frustration that is inevitable in the therapeutic setting (McWilliams, 2004). Thus, in light of the considerations discussed above, we might assume that clinicians’ ability to make accurate clinical decisions (or predictions) in psychotherapy relates to neurophysiological processes that mainly involve the hippocampus.

Despite the reported strengths and implications for future research, the present study has some notable limitations, including the relatively small number of recruited therapists. As regards this point, we wish to underline that the research is ongoing, and the recruitment of additional cases is underway. A robust enlargement of the therapist sample will allow us to investigate how, in clinicians, therapeutic reactions mediate neural responses during the observation of patient faces. An additional limitation is that distractors (i.e., unfamiliar faces) were not considered in the analyses. We are aware that the inclusion of distractors as control variables would have clarified (and perhaps supported) the above considerations. This is what we intend to verify in the next study.

Bearing in mind these limitations, to the best of our knowledge, no previous study has investigated therapist response patterns to patients with various personality pathologies from a neuroscience perspective. The results of the study, albeit preliminary, hold promise for the field, shedding light on the role played by therapists’ memory processes in clinical practice.

## Data availability statement

The original contributions presented in this study are included in the article, further inquiries can be directed to the corresponding author.

## Ethics statement

The studies involving human participants were reviewed and approved by Ethical Committee of the Department of Dynamic, Clinical Psychology, and Health Studies, Sapienza University of Rome, Italy. The patients/participants provided their written informed consent to participate in this study.

## Author contributions

AT conceived the study and wrote clinical issues of the entire manuscript. As first author, she was primarily accountable for all aspects of the work. CT wrote neural issues of the Introduction, Discussion, and Conclusions of the manuscript. AG performed ML analyses, wrote ML results, and provided a substantial contribution to the interpretation of hdEEG data. NC wrote the Introduction section and contributed substantially to the interpretation of the clinical aspects of the study. CC acquired hdEEG data and wrote “hdEEG recordings and data processing” section. CL designed the hdEEG study, monitored hdEEG data acquisition, and wrote “hdEEG recordings and data processing” section. MS-R contributed to the hdEEG study. VL contributed substantially to the conception, design of the work, drafted the manuscript, revised it for intellectual content, and approved its final version to be published. All authors agreed to ensure that the questions that were related to the accuracy or integrity of any part of the work were appropriately investigated and resolved.

## References

[B1] Adams R. B. Jr. Kleck R. E. (2003). Perceived gaze direction and the processing of facial displays of emotion. *Psychol. Sci.* 14 644–647. 10.1046/j.0956-7976.2003.psci_1479.x 14629700

[B2] Adams R. B. Jr. Kleck R. E. (2005). Effects of direct and averted gaze on the perception of facially communicated emotion. *Emotion* 5 3–11. 10.1037/1528-3542.5.1.3 15755215

[B3] Addis D. R. Moscovitch M. Crawley A. P. McAndrews M. P. (2004). Recollective qualities modulate hippocampal activation during autobiographical memory retrieval. *Hippocampus* 14 752–762. 10.1002/hipo.20405 15318333

[B4] Addis D. R. Schacter D. L. (2008). Constructive episodic simulation: temporal distance and detail of past and future events modulate hippocampal engagement. *Hippocampus* 18 227–237. 18157862 10.1002/hipo.20405

[B5] Addis D. R. Wong A. T. Schacter D. L. (2007). Remembering the past and imagining the future: common and distinct neural substrates during event construction and elaboration. *Neuropsychologia* 45 1363–1377. 10.1016/j.neuropsychologia.2006.10.016 17126370 PMC1894691

[B6] Allison T. Puce A. Spencer D. D. McCarthy G. (1999). Electrophysiological studies of human face perception: I. potentials generated in occipitotemporal cortex by face and non-face stimuli. *Cereb. Cortex* 9 415–430. 10.1093/cercor/9.5.415 10450888

[B7] Altavilla D. Ciacchella C. Pellicano G. R. Cecchini M. Tambelli R. Kalsi N. (2021). Neural correlates of sex-related differences in attachment dimensions. *Cogn. Affect. Behav. Neurosci.* 21 191–211. 10.3758/s13415-020-00859-5 33560494 PMC7994245

[B8] American Psychiatric Association [APA] (1994). *Diagnostic and Statistical Manual of Mental Disorders*, 4th Edn. Washington, DC: American Psychiatric Association Publishing.

[B9] Artuso C. Palladino P. Ricciardelli P. (2021). Memory updating through aging: different patterns for socially meaningful (and not) stimuli. *Aging Clin. Exp. Res.* 33 1005–1013. 10.1007/s40520-020-01604-1 32500367

[B10] Azizian A. Polich J. (2007). Evidence for attentional gradient in the serial position memory curve from event-related potentials. *J. Cogn. Neurosci.* 19 2071–2081. 10.1162/jocn.2007.19.12.2071 17892393 PMC2748728

[B11] Barron H. C. Auksztulewicz R. Friston K. (2020). Prediction and memory: a predictive coding account. *Prog. Neurobiol.* 192:101821. 10.1016/j.pneurobio.2020.101821 32446883 PMC7305946

[B12] Bateman A. Fonagy P. (2016). *Mentalization-based Treatment for Personality Disorders: A Practical Guide.* Oxford: Oxford University Press, 10.1093/med:psych/9780199680375.001.0001

[B13] Bateman A. W. Fonagy P. (2006). *Mentalization Based Treatment for Borderline Personality Disorder: A Practical Guide.* New York, NY: Oxford University Press.

[B14] Beck A. T. Davis D. D. Freeman A. (2004). *Cognitive Therapy of Personality Disorders*, 2nd Edn. New York, NY: Guilford Press.

[B15] Begleiter H. Porjesz B. Garozzo R. (1979). “Visual evoked potentials and affective ratings of semantic stimuli,” in *Evoked Brain Potentials and Behavior*, ed. Begleiter H. (Boston, MA: Springer), 127–141.

[B16] Berg J. Lundh L. G. Falkenstrom F. (2019). Countertransference in Swedish psychotherapists: testing the factor structure of the therapist response questionnaire. *Res. Psychother.* 22:331. 10.4081/ripppo.2019.331 32913777 PMC7453160

[B17] Betan E. Heim A. K. Zittel Conklin C. Westen D. (2005). Countertransference phenomena and personality pathology in clinical practice: an empirical investigation. *Am. J. Psychiatry* 162 890–898. 10.1176/appi.ajp.162.5.890 15863790

[B18] Blagov P. S. Bi W. Shedler J. Westen D. (2012). The Shedler-Westen Assessment Procedure (SWAP): evaluating psychometric questions about its reliability, validity, and impact of its fixed score distribution. *Assessment* 19 370–382. 10.1177/1073191112436667 22327208

[B19] Blatt S. J. (2004). *Experiences of Depression: Theoretical, Clinical, and Research Perspectives.* Washington, DC: American Psychological Association, 10.1037/10749-000

[B20] Blatt S. J. Shahar G. (2004). Psychoanalysis–with whom, for what, and how? comparisons with psychotherapy. *J. Am. Psychoanal. Assoc.* 52 393–447. 10.1177/00030651040520020401 15222434

[B21] Boston Change Process Study Group (2010). *Change in Psychotherapy: A Unifying Paradigm.* New York, NY: W. W. Norton and Company. 10.1176/appi.ajp.2010.10060873 26649788

[B22] Bourisly A. K. Shuaib A. (2018). Sex differences in electrophysiology: P200 event-related potential evidence. *Transl. Neurosci.* 9 72–77. 10.1515/tnsci-2018-0013 29967692 PMC6024693

[B23] Bourke M. E. Grenyer B. F. (2013). Therapists’ accounts of psychotherapy process associated with treating patients with borderline personality disorder. *J. Pers. Disord.* 27 735–745. 10.1521/pedi_2013_27_108 23718787

[B24] Bradley M. M. (2009). Natural selective attention: orienting and emotion. *Psychophysiology* 46 1–11. 10.1111/j.1469-8986.2008.00702.x 18778317 PMC3645482

[B25] Bush G. Phan L. Posner M. I. (2000). Cognitive and emotional influences in anterior cingulate cortex. *Trends Cogn. Sci.* 4 215–222. 10.1016/s1364-6613(00)01483-2 10827444

[B26] Caligor E. Levy K. N. Yeomans F. E. (2015). Narcissistic personality disorder: diagnostic and clinical challenges. *Am. J. Psychiatry* 172 415–422. 10.1176/appi.ajp.2014.14060723 25930131

[B27] Carretié L. Hinojosa J. A. López-Martín S. Tapia M. (2007). An electrophysiological study on the interaction between emotional content and spatial frequency of visual stimuli. *Neuropsychologia* 45 1187–1195. 10.1016/j.neuropsychologia.2006.10.013 17118408

[B28] Carretié L. Mercado F. Hinojosa J. A. Martın-Loeches M. Sotillo M. (2004). Valence-related vigilance biases in anxiety studied through event-related potentials. *J. Affect. Disord.* 78 119–130. 10.1016/s0165-0327(02)00242-2 14706722

[B29] Cecchini M. Aceto P. Altavilla D. Palumbo L. Lai C. (2013). The role of the eyes in processing an intact face and its scrambled image: a dense array ERP and low-resolution electromagnetic tomography (sLORETA) study. *Soc. Neurosci.* 8 314–325. 10.1080/17470919.2013.797020 23706064

[B30] Chertoff J. Kulish N. Levinson N. Schuker E. (2020). “Gender issues in transference and countertransference,” in *Female psychology: An annotated psychoanalytic bibliography* (New York, NY: Routledge).

[B31] Clarkin J. F. Yeomans F. E. Kernberg O. F. (2006). *Psychotherapy for Borderline Personality: Focusing on Object Relations.* Washington, DC: American Psychiatric Association Publishing.

[B32] Clemence A. J. Perry J. C. Plakun E. M. (2009). Narcissistic and borderline personality disorders in a sample of treatment refractory patients. *Psychiatr. Ann.* 39 175–184. 10.3928/00485713-20090401-05

[B33] Cohen N. J. Eichenbaum H. (1995). *Memory, Amnesia, and the Hippocampal System.* Cambridge, MA: MIT Press.

[B34] Cohen N. J. Ryan J. Hunt C. Romine L. Wszalek T. Nash C. (1999). Hippocampal system and declarative (relational) memory: summarizing the data from functional neuroimaging studies. *Hippocampus* 9 83–98. 10.1002/(SICI)1098-1063(1999)9:1<83::AID-HIPO9>3.0.CO;2-7 10088903

[B35] Colli A. Tanzilli A. Dimaggio G. Lingiardi V. (2014). Patient personality and therapist response: an empirical investigation. *Am. J. Psychiatry* 171 102–108. 10.1176/appi.ajp.2013.13020224 24077643

[B36] Conley E. M. Michalewski H. J. Starr A. (1999). The N100 auditory cortical evoked potential indexes scanning of auditory short-term memory. *Clin. Neurophysiol.* 110 2086–2093. 10.1016/s1388-2457(99)00183-2 10616113

[B37] Conley M. I. Dellarco D. V. Rubien-Thomas E. Cohen A. O. Cervera A. Tottenham N. (2018). The racially diverse affective expression (RADIATE) face stimulus set. *Psychiatry Res.* 270 1059–1067. 10.1016/j.psychres.2018.04.066 29910020 PMC6446554

[B38] Craik F. I. Moroz T. M. Moscovitch M. Stuss D. T. Winocur G. Tulving E. (1999). In search of the self: a positron emission tomography study. *Psychol. Sci.* 10 26–34. 10.1111/1467-9280.00102

[B39] Crisp H. Gabbard G. O. (2020). Principles of psychodynamic treatment for patients with narcissistic personality disorder. *J. Pers. Disord.* 34(Suppl.), 143–158. 10.1521/pedi.2020.34.supp.143 32186987

[B40] Critchley H. D. (2004). The human cortex responds to an interoceptive challenge. *Proc. Natl. Acad. Sci. U S A.* 101 6333–6334. 10.1073/pnas.0401510101 15096592 PMC404044

[B41] Cuthbert B. N. Schupp H. T. Bradley M. M. Birbaumer N. Lang P. J. (2000). Brain potentials in affective picture processing: covariation with autonomic arousal and affective report. *Biol. Psychol.* 52 95–111. 10.1016/s0301-0511(99)00044-7 10699350

[B42] Dadomo H. Salvato G. Lapomarda G. Ciftci Z. Messina I. Grecucci A. (2022). Structural features predict sexual trauma and interpersonal problems in borderline personality disorder but not in controls: A multi-voxel pattern analysis. *Front. Hum. Neurosci.* 16:773593. 10.3389/fnhum.2022.773593 35280205 PMC8904389

[B43] Dahl H. S. Røssberg J. I. Bøgwald K. P. Gabbard G. O. Høglend P. A. (2012). Countertransference feelings in one year of individual therapy: an evaluation of the factor structure in the feeling word Checklist-58. *Psychother. Res.* 22 12–25. 10.1080/10503307.2011.622312 22040366

[B44] Dahl H.-S. J. Røssberg J. I. Crits-Christoph P. Gabbard G. O. Hersoug A. G. Perry J. C. (2014). Long-term effects of analysis of the patient-therapist relationship in the context of patients’ personality pathology and therapists’ parental feelings. *J. Consult. Clin. Psychol.* 82 460–471. 10.1037/a0036410 24660675

[B45] Daselaar S. M. Rice H. J. Greenberg D. L. Cabeza R. LaBar K. S. Rubin D. C. (2008). The spatiotemporal dynamics of autobiographical memory: neural correlates of recall, emotional intensity, and reliving. *Cereb. Cortex* 18 217–229. 10.1093/cercor/bhm048 17548799

[B46] Diéguez-Risco T. Aguado L. Albert J. Hinojosa J. A. (2013). Faces in context: modulation of expression processing by situational information. *Soc. Neurosci.* 8 601–620. 10.1080/17470919.2013.834842 24053118

[B47] Diéguez-Risco T. Aguado L. Albert J. Hinojosa J. A. (2015). Judging emotional congruency: explicit attention to situational context modulates processing of facial expressions of emotion. *Biol. Psychol.* 112 27–38. 10.1016/j.biopsycho.2015.09.012 26450006

[B48] Dolcos F. Cabeza R. (2002). Event-related potentials of emotional memory: encoding pleasant, unpleasant, and neutral pictures. *Cogn. Affect. Behav. Neurosci.* 2 252–263. 10.3758/cabn.2.3.252 12775189

[B49] Dolcos F. Denkova E. Dolcos S. (2012). Neural correlates of emotional memories: a review of evidence from brain imaging studies. *Psychologia* 55 80–111. 10.2117/psysoc.2012.80

[B50] Dunning J. P. Hajcak G. (2009). See no evil: directing visual attention within unpleasant images modulates the electrocortical response. *Psychophysiology* 46 28–33. 10.1111/j.1469-8986.2008.00723.x 18992071

[B51] Eichenbaum H. (2001). The hippocampus and declarative memory: cognitive mechanisms and neural codes. *Behav. Brain Res.* 127 199–207. 11718892 10.1016/s0166-4328(01)00365-5

[B52] Eimer M. Holmes A. (2007). Event-related brain potential correlates of emotional face processing. *Neuropsychologia* 45 15–31. 10.1016/j.neuropsychologia.2006.04.022 16797614 PMC2383989

[B53] Eimer M. Holmes A. McGlone F. P. (2003). The role of spatial attention in the processing of facial expression: an ERP study of rapid brain responses to six basic emotions. *Cogn. Affect. Behav. Neurosci.* 3 97–110. 10.3758/cabn.3.2.97 12943325

[B54] Ekstrom S. R. (2014). *Memory and Healing: Neurocognitive and Psychodynamic Perspectives on how Patients and Psychotherapists Remember.* London: Karnac. 10.1111/1468-5922.12136_5 29749601

[B55] Eldridge L. L. Knowlton B. J. Furmanski C. S. Bookheimer S. Y. Engel S. A. (2000). Remembering episodes: a selective role for the hippocampus during retrieval. *Nat. Neurosci.* 3 1149–1152. 10.1038/80671 11036273

[B56] Falkenstein M. Hoormann J. Hohnsbein J. (1999). ERP components in Go/Nogo tasks and their relation to inhibition. *Acta Psycholog.* 101 267–291. 10.1016/s0001-6918(99)00008-6 10344188

[B57] Felleman D. J. Van Essen D. C. (1991). Distributed hierarchical processing in the primate cerebral cortex. *Cereb. Cortex* 1 1–47. 10.1093/cercor/1.1.1-a 1822724

[B58] Foti D. Hajcak G. (2008). Deconstructing reappraisal: descriptions preceding arousing pictures modulate the subsequent neural response. *J. Cogn. Neurosci.* 20 977–988. 10.1162/jocn.2008.20066 18211235

[B59] Foti D. Hajcak G. Dien J. (2009). Differentiating neural responses to emotional pictures: evidence from temporal-spatial PCA. *Psychophysiology* 46 521–530. 10.1111/j.1469-8986.2009.00796.x 19496228

[B60] Frenkel T. I. Bar-Haim Y. (2011). Neural activation during the processing of ambiguous fearful facial expressions: an ERP study in anxious and nonanxious individuals. *Biol. Psychol.* 88 188–195. 10.1016/j.biopsycho.2011.08.00 21846487

[B61] Freud S. (1910). “The future prospects of psychoanalytic therapy,” in *The Standard Edition of the Complete Psychological Works of Sigmund Freud*, ed. Strachey J. (Richmond, LDN: Hogarth Press), 139–151.

[B62] Freud S. (1912). “Recommendations to physicians practicing psychoanalysis,” in *The Standard Edition of the Complete Psychological Works of Sigmund Freud*, ed. Strachey J. (Richmond, LDN: Hogarth Press), 111–120.

[B63] Gabbard G. O. (1995). Countertransference: the emerging common ground. *Int. J. Psychoanal.* 76 475–485. 7558607

[B64] Gabbard G. O. (2009a). *Textbook of Psychotherapeutic Treatments.* Washington, D.C: American Psychiatric Association Publishing.

[B65] Gabbard G. O. (2009b). Transference and countertransference: developments in the treatment of narcissistic personality disorder. *Psychiatr. Ann.* 39 129–136. 10.3928/00485713-20090301-03

[B66] Gabbard G. O. (2014). *Gabbard’s Treatments of Psychiatric Disorders*, 5th Edn. Arlington, VA: American Psychiatric Publishing.

[B67] Gabbard G. O. Westen D. (2003). Rethinking therapeutic action. *Int. J. Psychoanal.* 84 823–841. 10.1516/002075703768284605 13678491

[B68] Gazzaniga M. S. (2008). *Human: The Science Behind What Makes Your Brain Unique.* New York, NY: Harper Perennial.

[B69] Ghani U. Signal N. Niazi I. K. Taylor D. (2020). ERP based measures of cognitive workload: a review. *Neurosci. Biobehav. Rev.* 118 18–26. 10.1016/j.neubiorev.2020.07.020 32707343

[B70] Gilboa A. (2004). Autobiographical and episodic memory–one and the same? evidence from prefrontal activation in neuroimaging studies. *Neuropsychologia* 42 1336–1349. 10.1016/j.neuropsychologia.2004.02.014 15193941

[B71] Grecucci A. Lapomarda G. Messina I. Monachesi B. Sorella S. Siugzdaite R. (2022). Structural features related to affective instability correctly classify the diagnosis of borderline personality disorder. A supervised machine learning approach. *Front. Psychiatry* 13:804440. 10.3389/fpsyt.2022.804440 35295769 PMC8918568

[B72] Grecucci A. Sulpizio S. Tommasello E. Vespignani F. Job R. (2018). Seeing emotions, reading emotions: behavioral and ERPs evidence of the effect of strategy and of regulation for pictures and words. *PLoS One* 14:e0209461. 10.1371/journal.pone.0209461 31150397 PMC6544208

[B73] Gusnard D. A. Akbudak E. Shulman G. L. Raichle M. E. (2001). Medial prefrontal cortex and self-referential mental activity: relation to a default mode of brain function. *Proc. Natl. Acad. Sci. U S A.* 98 4259–4264. 10.1073/pnas.071043098 11259662 PMC31213

[B74] Hajcak G. Dunning J. P. Foti D. (2009). Motivated and controlled attention to emotion: time-course of the late positive potential. *Clin. Neurophysiol.* 120 505–510. 10.1016/j.clinph.2008.11.028 19157974

[B75] Hajcak G. MacNamara A. Olvet D. M. (2010). Event-related potentials, emotion, and emotion regulation: an integrative review. *Dev. Neuropsychol.* 35 129–155. 10.1080/87565640903526504 20390599

[B76] Hajcak G. Olvet D. M. (2008). The persistence of attention to emotion: brain potentials during and after picture presentation. *Emotion* 8 250–255. 10.1037/1528-3542.8.2.250 18410198

[B77] Hayes J. A. Gelso C. J. Goldberg S. Kivlighan D. M. (2018). Countertransference management and effective psychotherapy: meta-analytic findings. *Psychotherapy* 55 496–507. 10.1037/pst0000189 30335461

[B78] Heimann P. (1950). On counter-transference. *Int. J. Psychoanal.* 31 81–84.

[B79] Hennissen V. C. Meganck R. Van Nieuwenhove K. Norman U. A. Loeys T. Desmet M. (2019). Therapists’ responses toward dependent (anaclitic) and self-critical (introjective) depressed outpatients: a multilevel approach. *Psychotherapy* 56 193–204. 10.1037/pst0000213 30869971

[B80] Hietanen J. K. Astikainen P. (2013). N170 response to facial expressions is modulated by the affective congruency between the emotional expression and preceding affective picture. *Biol. Psychol.* 92 114–124. 10.1016/j.biopsycho.2012.10.005 23131616

[B81] Holmes A. Nielsen M. K. Green S. (2007). Effects of anxiety on the processing of fearful and happy faces: An event-related potential study. *Biol. Psychol.* 77 159–173. 10.1016/j.biopsycho.2007.10.003 18022310

[B82] Hung Y. Smith M. L. Bayle D. J. Mills T. Cheyne D. Taylor M. J. (2010). Unattended emotional faces elicit early lateralized amygdala-frontal and fusiform activations. *Neuroimage* 50 727–733. 10.1016/j.neuroimage.2009.12.093 20045736

[B83] Jatoi M. A. Kamel N. Malik A. S. Faye I. (2014). EEG based brain source localization comparison of sLORETA and eLORETA. *Australas. Phys. Eng. Sci. Med.* 37 713–721. 10.1007/s13246-014-0308-3 25359588

[B84] Johnson S. C. Baxter L. C. Wilder L. S. Pipe J. G. Heiserman J. E. Prigatano G. P. (2002). Neural correlates of self-reflection. *Brain* 125 1808–1814. 10.1093/brain/awf181 12135971

[B85] Kanske P. Kotz S. A. (2007). Concreteness in emotional words: ERP evidence from a hemifield study. *Brain Res.* 1148 138–148. 10.1016/j.brainres.2007.02.044 17391654

[B86] Keil A. Müller M. M. Gruber T. Wienbruch C. Stolarova M. Elbert T. (2001). Effects of emotional arousal in the cerebral hemispheres: a study of oscillatory brain activity and event-related potentials. *Clin. Neurophysiol.* 112 2057–2068. 10.1016/s1388-2457(01)00654-11682344

[B87] Kernberg O. (1965). Notes on counter-transference. *J. Am. Psychoanal. Assoc.* 13 38–56. 10.1177/000306516501300102 14261795

[B88] Kernberg O. F. (1975). *Borderline Conditions and Pathological Narcissism.* Northvale, NJ: Aronson.

[B89] Kernberg O. F. (2014). An overview of the treatment of severe narcissistic pathology. *Int. J. Psychoanal.* 95 865–888. 10.1111/1745-8315.12204 24902768

[B90] Kissler J. Assadollahi R. Herbert C. (2006). Emotional and semantic networks in visual word processing: insights from ERP studies. *Prog. Brain Res.* 156 147–183. 10.1016/S0079-6123(06)56008-X 17015079

[B91] Klein F. Iffland B. Schindler S. Wabnitz P. Neuner F. (2015). This person is saying bad things about you: the influence of physically and socially threatening context information on the processing of inherently neutral faces. *Cogn. Affect. Behav. Neurosci.* 15 736–748. 10.3758/s13415-015-0361-8 25967930

[B92] Lai C. Pellicano G. R. Ciacchella C. Guidobaldi L. Altavilla D. Cecchini M. (2020). Neurophysiological correlates of emotional face perception consciousness. *Neuropsychologia* 146:107554. 10.1016/j.neuropsychologia.2020.107554 32652090

[B93] Lancaster J. L. Woldorff M. G. Parsons L. M. Liotti M. Freitas C. S. Rainey L. (2000). Automated talairach atlas labels for functional brain mapping. *Hum. Brain Mapp.* 10 120–131. 10912591 10.1002/1097-0193(200007)10:3<120::AID-HBM30>3.0.CO;2-8 PMC6871915

[B94] Lane R. D. Ryan L. Nadel L. Greenberg L. (2015). Memory reconsolidation, emotional arousal, and the process of change in psychotherapy: New insights from brain science. *Behav. Brain Sci.* 38:e1. 10.1017/S0140525X14000041 24827452

[B95] Ligeza T. S. Maciejczyk M. Kałamała P. Szygula Z. Wyczesany M. (2018). Moderate-intensity exercise boosts the N2 neural inhibition marker: a randomized and counterbalanced ERP study with precisely controlled exercise intensity. *Biol. Psychol.* 135 170–179. 10.1016/j.biopsycho.2018.04.003 29665432

[B96] Lingiardi V. McWilliams N. (eds) (2017). *Psychodynamic Diagnostic Manual (PDM-2)*, 2nd Edn. New York, NY: Guilford Press.

[B97] Lingiardi V. McWilliams N. (2015). The psychodynamic diagnostic manual, 2nd ed. (PDM-2). *World Psychiatry* 14 237–239. 10.1002/wps.20233 26043343 PMC4471982

[B98] Lingiardi V. Tanzilli A. Colli A. (2015). Does the severity of psychopathological symptoms mediate the relationship between patient personality and therapist response? *Psychotherapy* 52 228–237. 10.1037/a0037919 25383652

[B99] Loh W. Y. (2011). Classification and regression trees. *Wiley Interdiscip. Rev. Data Min. Knowl. Discov.* 1 14–23. 10.1002/widm.8 PMC3329156 22523608

[B100] Luck S. (2014). “Event-related potentials,” in *APA Handbook of Research Methods in Psychology: Foundations, Planning, Measures, and Psychometrics*, eds Cooper H. Camic P. M. Long D. L. Panter A. Rindskopf D. Sher K. J. (Washington, D.C: American Psychological Association), 1–9.

[B101] Luck S. J. (2005). *An Introduction to the Event-Related Potential Technique.* Cambridge, MA: MIT Press.

[B102] Luck S. J. Hillyard S. A. (1994). Electrophysiological correlates of feature analysis during visual search. *Psychophysiology* 31 291–308. 10.1111/j.1469-8986.1994.tb02218.x 8008793

[B103] MacNamara A. Hajcak G. (2009). Anxiety and spatial attention moderate the electrocortical response to aversive pictures. *Neuropsychologia* 47 2975–2980. 10.1016/j.neuropsychologia.2009.06.026 19576234

[B104] MacNamara A. Hajcak G. (2010). Distinct electrocortical and behavioral evidence for increased attention to threat in generalized anxiety disorder. *Depress. Anxiety* 27 234–243. 10.1002/da.20679 20196100

[B105] Maguire E. A. (2001). Neuroimaging studies of autobiographical event memory. *Philos. Trans. R. Soc. Lond. B. Biol. Sci.* 356 1441–1451. 10.1098/rstb.2001.0944 11571035 PMC1088527

[B106] Maguire E. A. Frith C. D. Cipolotti L. (2001). Distinct neural systems for the encoding and recognition of topography and faces. *Neuroimage* 13 743–750. 10.1006/nimg.2000.0712 11305901

[B107] Massaro G. Altavilla D. Aceto P. Pellicano G. R. Lucarelli G. Luciani M. (2018). Neurophysiological correlates of collective trauma recall in 2009 L’Aquila earthquake survivors. *J. Trauma. Stress.* 31 687–697. 10.1002/jts.22334 30338570

[B108] McIntyre S. M. Schwartz R. C. (1998). Therapists’ differential countertransference reactions toward clients with major depression or borderline personality disorder. *J. Clin. Psychol.* 54 923–931. 9811129 10.1002/(sici)1097-4679(199811)54:7<923::aid-jclp6>3.0.co;2-f

[B109] McWilliams N. (2004). *Psychoanalytic Psychotherapy: A Practitioner’s Guide.* New York, NY: Guilford Press.

[B110] McWilliams N. Shedler J. (2017). “Personality Syndromes—P Axis,” in *Psychodynamic Diagnostic Manual, (PDM-2)*, eds Lingiardi V. McWilliams N. (New York, NY: Guilford Press), 15–74.

[B111] Mecklinger A. Pfeifer E. (1996). Event-related potentials reveal topographical and temporal distinct neuronal activation patterns for spatial and object working memory. *Cogn. Brain Res.* 4 211–224. 10.1016/s0926-6410(96)00034-1 8924049

[B112] Meeren H. K. van Heijnsbergen C. C. de Gelder B. (2005). Rapid perceptual integration of facial expression and emotional body language. *Proc. Natl. Acad. Sci. U S A.* 102 16518–16523. 10.1073/pnas.0507650102 16260734 PMC1283446

[B113] Mesulam M. M. (2013). Cholinergic circuitry of the human nucleus basalis and its fate in Alzheimer’s disease. *J. Comp. Neurol.* 521 4124–4144. 10.1002/cne.23415 23852922 PMC4175400

[B114] Mishkin M. Vargha-Khadem F. Gadian D. G. (1998). Amnesia and the organization of the hippocampal system. *Hippocampus* 8 212–216. 9662136 10.1002/(SICI)1098-1063(1998)8:3<212::AID-HIPO4>3.0.CO;2-L

[B115] Montaldi D. Mayes A. R. (2010). The role of recollection and familiarity in the functional differentiation of the medial temporal lobes. *Hippocampus* 20 1291–1314. 10.1002/hipo.20853 20928828

[B116] Moscovitch D. A. McAndrews M. P. (2002). Material-specific deficits in “remembering” in patients with unilateral temporal lobe epilepsy and excisions. *Neuropsychologia* 40 1335–1342. 10.1016/s0028-3932(01)00213-5 11931936

[B117] Mueller E. M. Hofmann S. G. Santesso D. L. Meuret A. E. Bitran S. Pizzagalli D. A. (2009). Electrophysiological evidence of attentional biases in social anxiety disorder. *Psychol. Med.* 39 1141–1152. 10.1017/S0033291708004820 19079826 PMC3204217

[B118] Mühlberger A. Wieser M. J. Herrmann M. J. Weyers P. Tröger C. Pauli P. (2009). Early cortical processing of natural and artificial emotional faces differs between lower and higher socially anxious persons. *J. Neural Transm.* 116 735–746. 10.1007/s00702-008-0108-6 18784899

[B119] Naumann E. Bartussek D. Diedrich O. Laufer M. (1992). Assessing cognitive and affective information processing functions of the brain by means of the late positive complex of the event-related potential. *J. Psychophysiol.* 6 285–298.

[B120] Nissen-Lie H. A. Dahl H. J. Høglend P. A. (2022). Patient factors predict therapists’ emotional countertransference differently depending on whether therapists use transference work in psychodynamic therapy. *Psychother. Res.* 32 3–15. 10.1080/10503307.2020.1762947 32404003

[B121] Norcross J. C. Lambert M. J. (eds) (2019). *Psychotherapy Relationships that Work: Evidence-based Therapist Contributions*, 3rd Edn. New York, NY: Oxford University Press.

[B122] Ogrodniczuk J. S. Kealy D. (2013). “Interpersonal problems of narcissistic patients,” in *Understanding and Treating Pathological Narcissism*, ed. Ogrodniczuk J. S. (Washington, DC: American Psychological Association), 113–127.

[B123] Okuda J. Fujii T. Ohtake H. Tsukiura T. Tanji K. Suzuki K. (2003). Thinking of the future and the past: the roles of the frontal pole and the medial temporal lobes. *Neuroimage* 19 1369–1380. 10.1016/s1053-8119(03)00179-4 12948695

[B124] Olofsson J. K. Nordin S. Sequeira H. Polich J. (2008). Affective picture processing: an integrative review of ERP findings. *Biol. Psychol.* 77 247–265. 10.1016/j.biopsycho.2007.11.006 18164800 PMC2443061

[B125] Olofsson J. K. Polich J. (2007). Affective visual event-related potentials: arousal, repetition, and time-on-task. *Biol. Psychol.* 75 101–108. 10.1016/j.biopsycho.2006.12.006 17275979 PMC1885422

[B126] Palomba D. Angrilli A. Mini A. (1997). Visual evoked potentials, heart rate responses and memory to emotional pictorial stimuli. *Int. J. Psychophysiol.* 27 55–67. 10.1016/s0167-8760(97)00751-4 9161892

[B127] Pandya D. N. Seltzer B. (1982). Association areas of the cerebral cortex. *Trends Neurosci.* 5 386–390. 10.1016/0166-2236(82)90219-3

[B128] Pascual-Marqui R. D. (2002). Standardized low-resolution brain electromagnetic tomography (sLORETA): technical details. *Methods Find. Exp. Clin. Pharmacol.* 24(Suppl. D), 5–12. 12575463

[B129] Pekel E. Özmen E. P. (2020). “Computational intelligence approach for classification of diabetes mellitus using decision tree,” in *Computational Intelligence and Soft Computing Aèpplications in Heathcare Management Science*, eds Gul M. Celik E. Mete S. Serin F. (Pennsylvania, PA: IGI Global), 87–103. 10.4018/978-1-7998-2581-4.ch005

[B130] Perry J. D. Perry J. C. (2004). Conflicts, defenses and the stability of narcissistic personality features. *Psychiatry* 27 310–330. 10.1521/psyc.67.4.310.56570 15801375

[B131] Peterson N. N. Schroeder C. E. Arezzo J. C. (1995). Neural generators of early cortical somatosensory evoked potentials in the awake monkey. *Electroencephalogr. Clin. Neurophysiol.* 96 248–260. 10.1016/0168-5597(95)00006-e 7750450

[B132] Picton T. W. Bentin S. Berg P. Donchin E. Hillyard S. A. Johnson R. (2000). Guidelines for using human event-related potentials to study cognition: recording standards and publication criteria. *Psychophysiology* 37 127–152. 10.1111/1469-8986.3720127 10731765

[B133] Pires L. Leitão J. Guerrini C. Simões M. R. (2014). Event-related brain potentials in the study of inhibition: cognitive control, source localization and age-related modulations. *Neuropsychol. Rev.* 24 461–490. 10.1007/s11065-014-9275-4 25407470

[B134] Polich J. (2007). Updating P300: an integrative theory of P3a and P3b. *Clin. Neurophysiol.* 118 2128–2148. 10.1016/j.clinph.2007.04.019 17573239 PMC2715154

[B135] Polyn S. M. Norman K. A. Kahana M. J. (2009). A context maintenance and retrieval model of organizational processes in free recall. *Psychol. Rev.* 116:129. 10.1037/a0014420 19159151 PMC2630591

[B136] Richardson M. P. Strange B. A. Dolan R. J. (2004). Encoding of emotional memories depends on amygdala and hippocampus and their interactions. *Nat. Neurosci.* 7 278–285. 10.1038/nn1190 14758364

[B137] Righart R. De Gelder B. (2006). Context influences early perceptual analysis of faces—an electrophysiological study. *Cereb. Cortex* 16 1249–1257. 10.1093/cercor/bhj066 16306325

[B138] Righart R. De Gelder B. (2008). Rapid influence of emotional scenes on encoding of facial expressions: an ERP study. *Soc. Cogn. Affect. Neurosci.* 3 270–278. 10.1093/scan/nsn021 19015119 PMC2566764

[B139] Rischer K. M. Savallampi M. Akwaththage A. Salinas Thunell N. Lindersson C. MacGregor O. (2020). In context: emotional intent and temporal immediacy of contextual descriptions modulate affective ERP components to facial expressions. *Soc. Cogn. Affect. Neurosci.* 15 551–560. 10.1093/scan/nsaa071 32440673 PMC7328032

[B140] Roche R. A. Dockree P. M. Garavan H. Foxe J. J. Robertson I. H. O’Mara S. M. (2004). EEG alpha power changes reflect response inhibition deficits after traumatic brain injury (TBI) in humans. *Neurosci. Lett.* 362 1–5. 10.1016/j.neulet.2003.11.064 15147767

[B141] Ronningstam E. (2012). Alliance building and narcissistic personality disorder. *J Clin Psychol.* 68 943–953. 10.1002/jclp.21898 22729478

[B142] Ronningstam E. (2016). Pathological narcissism and narcissistic personality disorder: recent research and clinical implications. *Curr. Behav. Neurosci. Rep.* 3 34–42.

[B143] Røssberg J. I. Karterud S. Pedersen G. Friis S. (2007). An empirical study of countertransference reactions toward patients with personality disorders. *Compr. Psychiatry* 48 225–230. 10.1016/j.comppsych.2007.02.002 17445515

[B144] Røssberg J. I. Karterud S. Pedersen G. Friis S. (2008). Specific personality traits evoke different countertransference reactions: an empirical study. *J. Nerv. Ment. Dis.* 196 702–708. 18791432 10.1097/NMD.0b013e318186de80

[B145] Rugg M. D. Vilberg K. L. (2013). Brain networks underlying episodic memory retrieval. *Curr. Opin. Neurobiol.* 23 255–260. 10.1016/j.conb.2012.11.005 23206590 PMC3594562

[B146] Sabatinelli D. Lang P. J. Keil A. Bradley M. M. (2007). Emotional perception: correlation of functional MRI and event-related potentials. *Cereb. Cortex* 17 1085–1091. 10.1093/cercor/bhl017 16769742

[B147] Schacter D. L. Addis D. R. (2007). The cognitive neuroscience of constructive memory: remembering the past and imagining the future. *Philos. Trans. R. Soc. Lond. B. Biol. Sci.* 362 773–786. 10.1098/rstb.2007.2087 17395575 PMC2429996

[B148] Schupp H. T. Cuthbert B. Bradley M. Hillman C. Hamm A. Lang P. (2004a). Brain processes in emotional perception: motivated attention. *Cogn. Emot.* 18 593–611. 10.1080/02699930341000239

[B149] Schupp H. T. Junghöfer M. Weike A. I. Hamm A. O. (2004b). The selective processing of briefly presented affective pictures: an ERP analysis. *Psychophysiology* 41 441–449. 10.1111/j.1469-8986.2004.00174.x 15102130

[B150] Schupp H. T. Cuthbert B. N. Bradley M. M. Cacioppo J. T. Ito T. Lang P. J. (2000). Affective picture processing: the late positive potential is modulated by motivational relevance. *Psychophysiology* 37 257–261. 10.1111/1469-8986.3720257 10731776

[B151] Schupp H. T. Flaisch T. Stockburger J. Junghöfer M. (2006). Emotion and attention: event-related brain potential studies. *Prog. Brain Res.* 156 31–51. 10.1016/S0079-6123(06)56002-9 17015073

[B152] Schwartz R. C. Smith S. D. Chopko B. (2007). Psychotherapists’ countertransference reactions toward clients with antisocial personality disorder and schizophrenia: an empirical test of theory. *Am. J. Psychother.* 61 375–393. 10.1176/appi.psychotherapy.2007.61.4.375 18251383

[B153] Scoville W. B. Milner B. (1957). Loss of recent memory after bilateral hippocampal lesions. *J. Neurol. Neurosurg. Psychiatry* 20 11–21. 10.1136/jnnp.20.1.11 13406589 PMC497229

[B154] Shedler J. Westen D. (2004). Dimensions of personality pathology: an alternative to the five-factor model. *Am. J. Psychiatry* 161 1743–1754. 10.1176/ajp.161.10.1743 15465966

[B155] Shedler J. Westen D. Lingiardi V. (2014). *The Evaluation of Personality with the SWAP-200.* Milan: Raffaello Cortina.

[B156] Sokhadze E. M. Casanova M. F. Casanova E. L. Lamina E. Kelly D. P. Khachidze I. (2017). Event-related potentials (ERP) in cognitive neuroscience research and applications. *Neuroregulation* 4 14–14. 10.15540/nr.4.1.14

[B157] Squire L. R. Stark C. E. Clark R. E. (2004). The medial temporal lobe. *Annu. Rev. Neurosci.* 27 279–306. 10.1146/annurev.neuro.27.070203.144130 15217334

[B158] Stefana A. Bulgari V. Youngstrom E. A. Dakanalis A. Bordin C. Hopwood C. J. (2020). Patient personality and psychotherapist reactions in individual psychotherapy setting: a systematic review. *Clin. Psychol. Psychother.* 27 697–713. 10.1002/cpp.2455 32251550

[B159] Stolz C. Endres D. Mueller E. M. (2019). Threat-conditioned contexts modulate the late positive potential to faces—a mobile EEG/virtual reality study. *Psychophysiology* 56:e13308. 10.1111/psyp.13308 30548599

[B160] Strange B. A. Dolan R. J. (2006). Anterior medial temporal lobe in human cognition: memory for fear and the unexpected. *Cogn. Neuropsychiatry* 11 198–218. 10.1080/13546800500305096 17354068 PMC2633117

[B161] Streiner D. L. (2003). Being inconsistent about consistency: when coefficient alpha does and doesn’t matter. *J. Pers. Assess.* 80 217–222. 10.1207/S15327752JPA8003_01 12763696

[B162] Sulpizio S. Toti M. Del Maschio N. Costa A. Fedeli D. Job R. (2019). Are you really cursing? neural processing of taboo words in native and foreign language. *Brain Lang.* 194 84–92. 10.1016/j.bandl.2019.05.003 31146214

[B163] Sur S. Sinha V. K. (2009). Event-related potential: an overview. *Ind. Psychiatry J.* 18 70–73. 10.4103/0972-6748.57865 21234168 PMC3016705

[B164] Talmi D. Lohnas L. J. Daw N. D. (2019). A retrieved context model of the emotional modulation of memory. *Psychol. Rev.* 126 455–485. 10.1037/rev0000132 30973247

[B165] Tanner D. Morgan-Short K. Luck S. J. (2015). How inappropriate high-pass filters can produce artifactual effects and incorrect conclusions in ERP studies of language and cognition. *Psychophysiology* 52 997–1009. 10.1111/psyp.12437 25903295 PMC4506207

[B166] Tanzilli A. Colli A. Del Corno F. Lingiardi V. (2016). Factor structure, reliability, and validity of the therapist response questionnaire. *Pers. Disord. Theory Res. Treat.* 7 147–158. 10.1037/per0000146 26389623

[B167] Tanzilli A. Gualco I. (2020). Clinician emotional responses and therapeutic alliance when treating adolescent patients with narcissistic personality disorder subtypes: A clinically meaningful empirical investigation. *J. Pers. Disord.* 34 42–62. 10.1521/pedi.2020.34.supp.42 32186983

[B168] Tanzilli A. Gualco I. Baiocco R. Lingiardi V. (2020). Clinician reactions when working with adolescent patients: the therapist response questionnaire for adolescents. *J. Pers. Assess.* 102 616–627. 10.1080/00223891.2019.1674318 31609644

[B169] Tanzilli A. Lingiardi V. Hilsenroth M. (2018). Patient SWAP-200 personality dimensions and FFM traits: do they predict therapist responses? *Pers. Disord. Theory Res. Treat.* 9 250–262. 10.1037/per0000260 28836804

[B170] Tanzilli A. Muzi L. Ronningstam E. Lingiardi V. (2017). Countertransference when working with narcissistic personality disorder: an empirical investigation. *Psychotherapy* 54 184–194. 10.1037/pst0000111 28581327

[B171] Van Essen D. C. Maunsell J. H. (1983). Hierarchical organization and functional streams in the visual cortex. *Trends Neurosci.* 6 370–375.

[B172] Vogel E. K. Luck S. J. (2000). The visual N1 component as an index of a discrimination process. *Psychophysiology* 37 190–203. 10.1111/1469-8986.3720190 10731769

[B173] Vuilleumier P. Pourtois G. (2007). Distributed and interactive brain mechanisms during emotion face perception: evidence from functional neuroimaging. *Neuropsychologia* 45 174–194. 16854439 10.1016/j.neuropsychologia.2006.06.003

[B174] Wampold B. E. (2015). How important are the common factors in psychotherapy? an update. *World Psychiatry* 14 270–277. 10.1002/wps.20238 26407772 PMC4592639

[B175] Weinberg A. Hajcak G. (2010). Beyond good and evil: the time-course of neural activity elicited by specific picture content. *Emotion* 10 767–782. 10.1037/a0020242 21058848

[B176] Weinberg A. Hajcak G. (2011). The late positive potential predicts subsequent interference with target processing. *J. Cogn. Neurosci.* 23 2994–3007. 10.1162/jocn.2011.21630 21268668

[B177] Wessing I. Rehbein M. A. Postert C. Fürniss T. Junghöfer M. (2013). The neural basis of cognitive change: reappraisal of emotional faces modulates neural source activity in a frontoparietal attention network. *Neuroimage* 81 15–25. 10.1016/j.neuroimage.2013.04.117 23664945

[B178] Westen D. Muderrisoglu S. (2006). Clinical assessment of pathological personality traits. *Am. J. Psychiatry* 163 1285–1287. 10.1176/ajp.2006.163.7.1285 16816238

[B179] Westen D. Shedler J. (1999a). Revising and assessing axis II, Part I: developing a clinically and empirically valid assessment method. *Am J Psychiatry* 156 258–272. 10.1176/ajp.156.2.258 9989563

[B180] Westen D. Shedler J. (1999b). Revising and assessing axis II, Part II: toward an empirically based and clinically useful classification of personality disorders. *Am. J. Psychiatry* 156 273–285. 10.1176/ajp.156.2.273 9989564

[B181] Wheeler M. A. Stuss D. T. Tulving E. (1997). Toward a theory of episodic memory: the frontal lobes and autonoetic consciousness. *Psychol. Bull.* 121 331–354. 10.1037/0033-2909.121.3.331 9136640

[B182] Wheeler M. E. Petersen S. E. Buckner R. L. (2000). Memory’s echo: vivid remembering reactivates sensory-specific cortex. *PNAS* 97 11125–11129. 10.1073/pnas.97.20.11125 11005879 PMC27159

[B183] Wieser M. J. Brosch T. (2012). Faces in context: a review and systematization of contextual influences on affective face processing. *Front. Psychol.* 3:471. 10.3389/fpsyg.2012.00471 23130011 PMC3487423

[B184] Witter M. P. (1993). Organization of the entorhinal—hippocampal system: a review of current anatomical data. *Hippocampus* 3 33–44. 8287110

[B185] Witter M. P. Groenewegen H. J. Lopes, da Silva F. H. Lohman A. H. (1989). Functional organization of the extrinsic and intrinsic circuitry of the parahippocampal region. *Prog. Neurobiol.* 33 161–253. 10.1016/0301-0082(89)90009-9 2682783

[B186] Xu M. Li Z. Diao L. Fan L. Yang D. (2016). Contextual valence and sociality jointly influence the early and later stages of neutral face processing. *Front. Psychol.* 7:1258. 10.3389/fpsyg.2016.01258 27594847 PMC4990723

[B187] Yeomans F. E. Clarkin J. F. Kernberg O. F. (2015). *Transference Focused Psychotherapy for Borderline Personality Disorder: A Clinical Guide.* Washington, DC: American Psychiatric Publishing.

[B188] Zittel Conklin C. Westen D. (2003). *The Therapist Response Questionnaire (TRQ).* Atlanta, GA: Departments of Psychology and Psychiatry and Behavioral Sciences, Emory University.

